# Supplementary material for: Worldwide Prevalence and Clinical Characteristics of RAS Mutations in Head and Neck Cancer: A Systematic Review and Meta-Analysis
Source: Front Oncol. 2022 May 6;12:838911. doi: 10.3389/fonc.2022.838911 (PMC9121358; doi:10.3389/fonc.2022.838911)
Supplement: Supplementary file 1 [file DataSheet_1.pdf]

## Supplementary Material


| | | |
|---|---|---|
| Page 1 | Supplementary Methods | Search strings |
| Page 2 | Figure S1 | Begg's funnel plots |
| Pages 3-7 | Table S1 | Literature references list of studies included in the meta-analysis |
| Pages 8-15 | Table S2 | Detailed characteristics of the studies included in the meta-analysis |
| Pages 16-31 | Table S3 | Detailed anatomical site data |
| | | Forest plot of RAS mutation frequency according to geographical region |
| | | Forest plot of RAS mutation frequency according to anatomical site |
| | | Association between RAS Mutations and Disease Stage/Grade |
| | | Association between RAS Mutations and HPV Status |


## Supplementary Methods: Search strings

A systematic literature review was conducted by searching the PubMed, Embase, Web of Science, and Cochrane Central Register of Controlled Trials databases in June 2021 for studies published in the English language since 1 January 2000. The search string included 'RAS' and 'mutation' and one of the following terms: 'Head and neck cancer', 'Head and neck squamous cell carcinoma', 'Oral cancer', 'oral squamous cell carcinoma', 'tongue', 'lips', 'nasopharyngeal'/'nasopharynx', 'pharyngeal'/'pharynx', 'laryngeal'/'larynx', 'oropharyngeal'/'oropharynx', 'Salivary gland', 'sinonasal'/'nasal'/'sinus', 'oropharyngeal'/'oropharynx', 'hypopharyngeal'/'hypopharynx', or 'tonsil'.

## Figure S1: Begg's funnel plots

Potential publication bias was analyzed by Begg's funnel plots, displaying the prevalence of mutations (x-axis) versus the standard error in each study (y-axis). Each dot represents a single study. The white rectangle indicates a 95% pseudo-confidence interval, and the solid middle line indicates the overall effect from the meta-analysis.

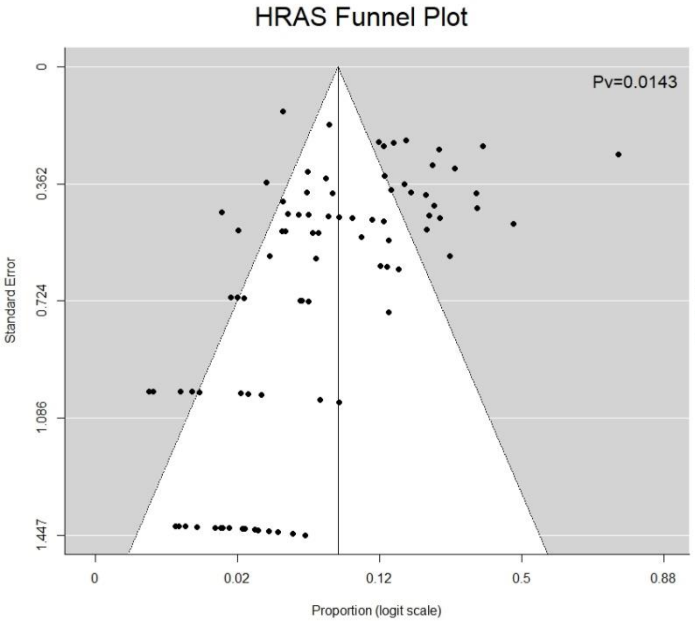


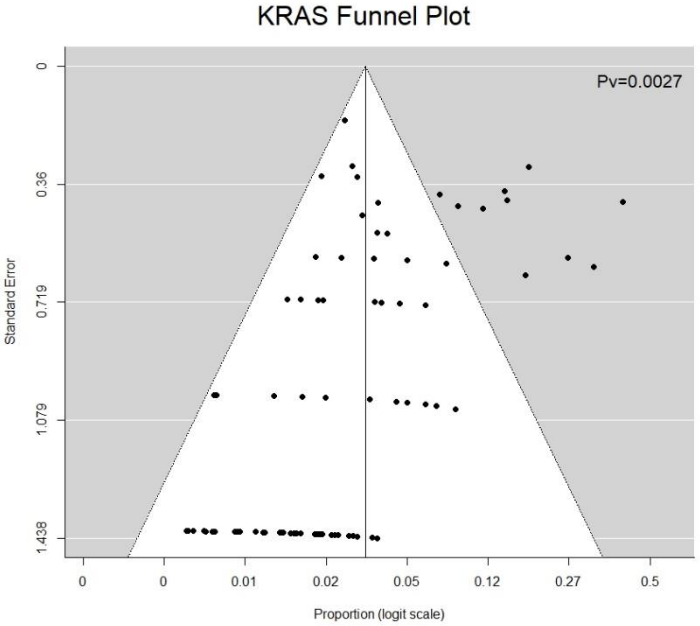


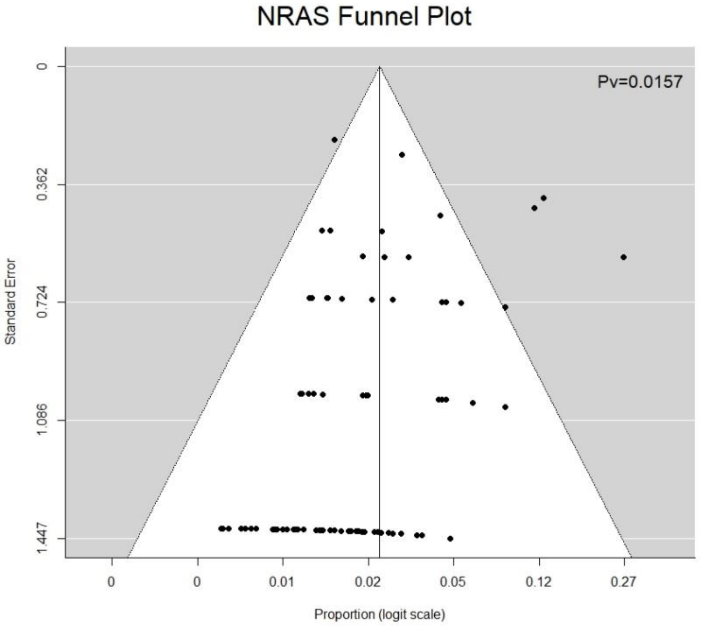

## Table S1: Literature references list of studies included in the meta-analysis


1. Das N, Majumder J, Dasgupta UB. ras Gene mutations in oral cancer in eastern India. Oral Oncol (2000) 36:76–80. doi:10.1016/S1368-8375(99)00058-5
2. Yoo J, Robinson RA. ras Gene Mutations in Salivary Gland Tumors. Arch Pathol Lab Med (2000) 124:836–839. doi:10.5858/2000-124-0836-RGMISG
3. Yoo J, Robinson RA. ras gene mutations in salivary gland tumors. Arch Pathol Lab Med (2000) 124:836–839. doi:10.5858/2000-124-0836-RGMISG
4. Lin S, Chen Z, Chen M-H, Wang H-M. [Express of human papillomavirus, P53 and H-ras gene in laryngeal cancer]. Zhejiang Da Xue Xue Bao Yi Xue Ban (2002) 31:231–234. doi:10.3785/j.issn.1008-9292.2002.04.002
5. Weber A, Langhanki L, Sommerer F, Markwarth A, Wittekind C, Tannapfel A. Mutations of the BRAF gene in squamous cell carcinoma of the head and neck. Oncogene (2003) 22:4757–4759. doi:10.1038/sj.onc.1206705
6. Perrone F, Oggionni M, Birindelli S, Suardi S, Tabano S, Romano R, Moiraghi ML, Bimbi G, Quattrone P, Cantu G, et al. TP53, P14ARF, P16INK4a and H-ras gene molecular analysis in intestinal-type adenocarcinoma of the nasal cavity and paranasal sinuses. Int J Cancer (2003) 105:196–203. doi:10.1002/ijc.11062
7. Ruíz-Godoy RLM, García-Cuellar CM, Herrera González NE, Suchil BL, Pérez-Cárdenas E, Sánchez-Pérez Y, Suárez-Roa ML, Meneses A. Mutational analysis of K-ras and Ras protein expression in larynx squamous cell carcinoma. J Exp Clin Cancer Res (2006) 25:73–78. Available at: https://europepmc.org/article/med/16761621 [Accessed September 12, 2021]
8. Sathyan KM, Nalinakumari KR, Abraham T, Kannan S. Influence of single nucleotide polymorphisms in H-Ras and cyclin D1 genes on oral cancer susceptibility. Oral Oncol (2006) 42:607–613. doi:10.1016/j.oraloncology.2005.10.019
9. Frattini M, Perrone F, Suardi S, Balestra D, Caramuta S, Colombo F, Licitra L, Cantù G, Pierotti MA, Pilotti S. Phenotype-genotype correlation: Challenge of intestinal-type adenocarcinoma of the nasal cavity and paranasal sinuses. Head Neck (2006) 28:909–915. doi:10.1002/hed.20433
10. Augello C, Gregorio V, Bazan V, Cammareri P, Agnese V, Cascio S, Corsale S, Calò V, Gullo A, Passantino R, et al. TP53 and P16INK4A, but not H-KI-RAS, are involved in tumorigenesis and progression of pleomorphic adenomas. J Cell Physiol (2006) 207:654–659. doi:10.1002/jcp.20601
11. Russo A, Corsale S, Agnese V, Macaluso M, Cascio S, Bruno L, Surmacz E, Dardanoni G, Valerio MR, Vieni S, et al. TP53 mutations and S-phase fraction but not DNA-ploidy are independent prognostic indicators in laryngeal squamous cell carcinoma. J Cell Physiol (2006) 206:181–188. doi:10.1002/jcp.20447
12. Sathyan KM, Nalinakumari KR, Kannan S. H-Ras mutation modulates the expression of major cell cycle regulatory proteins and disease prognosis in oral carcinoma. Mod Pathol (2007) 20:1141–1148. doi:10.1038/modpathol.3800948
13. Gupta R, Singh S, Hedau S, Nigam S, Das BC, Singh I, Mandal AK. Spindle cell carcinoma of head and neck: An immunohistochemical and molecular approach to its pathogenesis. J Clin Pathol (2007) 60:472–475. doi:10.1136/jcp.2005.033589
14. Sheikh Ali MAL, Gunduz M, Nagatsuka H, Gunduz E, Cengiz B, Fukushima K, Beder LB, Demircan K, Fujii M, Yamanaka N, et al. Expression and mutation analysis of epidermal growth factor receptor in head and neck squamous cell carcinoma. Cancer Sci (2008) 99:1589–1594. doi:10.1111/j.1349-7006.2008.00861.x
15. Chou CC, Chou MJ, Tzen CY. PIK3CA mutation occurs in nasopharyngeal carcinoma but does not significantly influence the disease-specific survival. Med Oncol (2009) 26:322–326. doi:10.1007/s12032-008-9124-5
16. Bornholdt J, Hansen J, Steiniche T, Dictor M, Antonsen A, Wolff H, Schlünssen V, Holmila R, Luce D, Vogel U, et al. K-ras mutations in sinonasal cancers in relation to wood dust exposure. BMC Cancer (2008) 8:1–11. doi:10.1186/1471-2407-8-53
17. Murugan AK, Thi Hong N, Fukui Y, Munirajan AK, Tsuchida N. Oncogenic mutations of the PIK3CA gene in head and neck squamous cell carcinomas. Int J Oncol (2008) 32:101–111. doi:10.3892/ijo.32.1.101
18. Dahse R, Driemel O, Schwarz S, Kromeyer-Hauschild K, Berndt A, Kosmehl H. KRAS status and epidermal growth factor receptor expression as determinants for anti-EGFR therapies in salivary gland carcinomas. Oral Oncol (2009) 45:826–829. doi:10.1016/j.oraloncology.2009.01.013
19. Murugan AK, Hong NT, Cuc TTK, Hung NC, Munirajan AK, Ikeda MA, Tsuchida N. Detection of two novel mutations and relatively high incidence of H-RAS mutations in Vietnamese oral cancer. Oral Oncol (2009) 45:e161–e166. doi:10.1016/j.oraloncology.2009.05.638
20. Bruckman KC, Schnleben F, Qiu W, Woo VL, Su GH. Mutational analyses of the BRAF, KRAS, and PIK3CA genes in oral squamous cell carcinoma. Oral Surgery, Oral Med Oral Pathol Oral Radiol Endodontology (2010) 110:632–637. doi:10.1016/j.tripleo.2010.05.002
21. Van Damme N, Deron P, Van Roy N, Demetter P, Bols A, Dorpe J Van, Baert F, Van Laethem JL, Speleman F, Pauwels P, et al. Epidermal Growth Factor Receptor and K-RAS status in two cohorts of squamous cell carcinomas. BMC Cancer (2010) 10: doi:10.1186/1471-2407-10-189
22. Chang YS, Yeh KT, Hsu NC, Lin SH, Chang TJ, Chang JG. Detection of N-, H-, and KRAS codons 12, 13, and 61 mutations with universal RAS primer multiplex PCR and N-, H-, and KRAS-specific primer extension. Clin Biochem (2010) 43:296–301. doi:10.1016/j.clinbiochem.2009.10.007
23. Tetsu O, Phuchareon J, Chou A, Cox DP, Eisele DW, Jordan RCK. Mutations in the c-Kit gene disrupt mitogen-activated protein kinase signaling during tumor development in adenoid cystic carcinoma of the salivary glands. Neoplasia (2010) 12:708–717. doi:10.1593/neo.10356
24. Murray S, Bobos M, Angouridakis N, Nikolaou A, Linardou H, Razis E, Fountzilas G. Screening for EGFR mutations in patients with head and neck cancer treated with gefitinib on a compassionate-use program: A Hellenic Cooperative Oncology group study. J Oncol (2010) 2010: doi:10.1155/2010/709678
25. Popović B, Jekić B, Novaković I, Luković L, Konstantinović V, Babić M, Milain J. Cancer genes alterations and HPV infection in oral squamous cell carcinoma. Int J Oral Maxillofac Surg (2010) 39:909–915. doi:10.1016/j.ijom.2010.05.007
26. Agrawal N, Frederick MJ, Pickering CR, Bettegowda C, Chang K, Li RJ, Fakhry C, Xie TX, Zhang J, Wang J, et al. Exome sequencing of head and neck squamous cell carcinoma reveals inactivating mutations in NOTCH1. Science (80- ) (2011) 333:1154–1157. doi:10.1126/SCIENCE.1206923
27. Stransky N, Egloff AM, Tward AD, Kostic AD, Cibulskis K, Sivachenko A, Kryukov G V., Lawrence MS, Sougnez C, McKenna A, et al. The mutational landscape of head and neck squamous cell carcinoma. Science (80- ) (2011) 333:1157–1160. doi:10.1126/science.1208130
28. Cohen Y, Goldenberg-Cohen N, Shalmon B, Shani T, Oren S, Amariglio N, Dratviman-Storobinsky O, Shnaiderman-Shapiro A, Yahalom R, Kaplan I, et al. Mutational analysis of PTEN/PIK3CA/AKT pathway in oral squamous cell carcinoma. Oral Oncol (2011) 47:946–950. doi:10.1016/j.oraloncology.2011.07.013
29. Wang WY, Chien YC, Wong YK, Lin YL, Lin JC. Effects of KRAS mutation and polymorphism on the risk and prognosis of oral squamous cell carcinoma. Head Neck (2012) 34:663–666. doi:10.1002/hed.21792

30. Friedland P, Thomas A, Naran A, Amanuel B, Grieu-Iacopetta F, Carrello A, Harnett G, Meyer C, Phillips M. Human papillomavirus and gene mutations in head and neck squamous carcinomas. ANZ J Surg (2012) 82:362–366. doi:10.1111/j.1445-2197.2011.05791.x
31. Szabó B, Nelhubel GA, Kárpáti A, Kenessey I, Jóri B, Székely C, Peták I, Lotz G, Hegedus Z, Hegedus B, et al. Clinical significance of genetic alterations and expression of epidermal growth factor receptor (EGFR) in head and neck squamous cell carcinomas. Oral Oncol (2011) 47:487–496. doi:10.1016/j.oraloncology.2011.03.020
32. Suda T, Hama T, Kondo S, Yuza Y, Yoshikawa M, Urashima M, Kato T, Moriyama H. Copy Number Amplification of the PIK3CA Gene Is Associated with Poor Prognosis in Non-lymph node metastatic Head and Neck Squamous Cell Carcinoma. BMC Cancer (2012) 12:416. doi:10.1186/1471-2407-12-416
33. Smilek P, Neuwirthova J, Jarkovsky J, Dusek L, Rottenberg J, Kostrica R, Srovnal J, Hajduch M, Drabek J, Klozar J. Epidermal growth factor receptor (EGFR) expression and mutations in the EGFR signaling pathway in correlation with anti-EGFR therapy in head and neck squamous cell carcinomas. Neoplasma (2012) 59:508–515. doi:10.4149/neo_2012_065
34. Koumaki D, Kostakis G, Koumaki V, Papadogeorgakis N, Makris M, Katoulis A, Kamakari S, Koutsodontis G, Perisanidis C, Lambadiari V, et al. Novel mutations of the HRAS gene and absence of hotspot mutations of the BRAF genes in oral squamous cell carcinoma in a Greek population. Oncol Rep (2012) 27:1555–1560. doi:10.3892/or.2012.1653
35. López F, García Inclán C, Pérez-Escuredo J, Álvarez Marcos C, Scola B, Suárez C, Llorente JL, Hermsen MA. KRAS and BRAF mutations in sinonasal cancer. Oral Oncol (2012) 48:692–697. doi:10.1016/j.oraloncology.2012.02.018
36. Bissada E, Abboud O, Abou Chacra Z, Guertin L, Weng X, Nguyen-Tan PF, Tabet J-C, Thibaudeau È, Lambert L, Audet M-L, et al. Prevalence of K-RAS Codons 12 and 13 Mutations in Locally Advanced Head and Neck Squamous Cell Carcinoma and Impact on Clinical Outcomes. Int J Otolaryngol (2013) 2013:1–6. doi:10.1155/2013/848021
37. Chiosea SI, Grandis JR, Lui VWY, Diergaarde B, Maxwell JH, Ferris RL, Kim SW, Luvison A, Miller M, Nikiforova MN. PIK3CA, HRAS and PTEN in human papillomavirus positive oropharyngeal squamous cell carcinoma. BMC Cancer (2013) 13: doi:10.1186/1471-2407-13-602
38. Lechner M, Frampton GM, Fenton T, Feber A, Palmer G, Jay A, Pillay N, Forster M, Cronin MT, Lipson D, et al. Targeted next-generation sequencing of head and neck squamous cell carcinoma identifies novel genetic alterations in HPV+ and HPV- tumors. Genome Med (2013) 5: doi:10.1186/gm453
39. Pickering CR, Zhang J, Yoo SY, Bengtsson L, Moorthy S, Neskey DM, Zhao M, Ortega Alves M V., Chang K, Drummond J, et al. Integrative genomic characterization of oral squamous cell carcinoma identifies frequent somatic drivers. Cancer Discov (2013) 3:770–781. doi:10.1158/2159-8290.CD-12-0537
40. Ho AS, Kannan K, Roy DM, Morris LGT, Ganly I, Katabi N, Ramaswami D, Walsh LA, Eng S, Huse JT, et al. The mutational landscape of adenoid cystic carcinoma. Nat Genet (2013) 45:791–798. doi:10.1038/ng.2643
41. Stephens PJ, Davies HR, Mitani Y, Van Loo P, Shlien A, Tarpey PS, Papaemmanuil E, Cheverton A, Bignell GR, Butler AP, et al. Whole exome sequencing of adenoid cystic carcinoma. J Clin Invest (2013) 123:2965–2968. doi:10.1172/JCI67201
42. Lui VWY, Hedberg ML, Li H, Vangara BS, Pendleton K, Zeng Y, Lu Y, Zhang Q, Du Y, Gilbert BR, et al. Frequent mutation of the PI3K pathway in head and neck cancer defines predictive biomarkers. Cancer Discov (2013) 3:761–769. doi:10.1158/2159-8290.CD-13-0103
43. Cros J, Sbidian E, Hans S, Roussel H, Scotte F, Tartour E, Brasnu D, Laurent-Puig P, Bruneval P, Blons H, et al. Expression and mutational status of treatment-relevant targets and key oncogenes in 123 malignant salivary gland tumours. Ann Oncol (2013) 24:2624–2629. doi:10.1093/annonc/mdt338
44. Fujii S, Uryu H, Akashi K, Suzuki K, Yamazaki M, Tahara M, Hayashi R, Ochiai A. Clinical significance of KRAS gene mutation and epidermal growth factor receptor expression in Japanese patients with squamous cell carcinoma of the larynx, oropharynx and hypopharynx. Int J Clin Oncol (2013) 18:454–463. doi:10.1007/s10147-012-0402-z
45. Projetti F, Durand K, Chaunavel A, Léobon S, Lacorre S, Caire F, Bessède JP, Moreau JJ, Coulibaly B, Labrousse F. Epidermal growth factor receptor expression and KRAS and BRAF mutations: Study of 39 sinonasal intestinal-type adenocarcinomas. Hum Pathol (2013) 44:2116–2125. doi:10.1016/j.humpath.2013.03.019
46. Szablewski V, Solassol J, Poizat F, Larrieux M, Crampette L, Mange A, Bascoul-Mollevi C, Costes V. EGFR expression and KRAS and BRAF mutational status in intestinal-type sinonasal adenocarcinoma. Int J Mol Sci (2013) 14:5170–5181. doi:10.3390/ijms14035170
47. De Carvalho TG, De Carvalho AC, Maia DCC, Ogawa JK, Carvalho AL, Vettore AL. Search for mutations in signaling pathways in head and neck squamous cell carcinoma. Oncol Rep (2013) 30:334–340. doi:10.3892/or.2013.2455
48. Zanaruddin SNS, Yee PS, Hor SY, Kong YH, Abd Ghani WMNW, Mustafa WMW, Zain RB, Prime SS, Abd Rahman ZA, Cheong SC. Common oncogenic mutations are infrequent in oral squamous cell carcinoma of Asian origin. PLoS One (2013) 8: doi:10.1371/journal.pone.0080229
49. Wetterskog D, Wilkerson PM, Rodrigues DN, Lambros MB, Fritchie K, Andersson MK, Natrajan R, Gauthier A, Palma S Di, Shousha S, et al. Mutation profiling of adenoid cystic carcinomas from multiple anatomical sites identifies mutations in the RAS pathway, but no KIT mutations. Histopathology (2013) 62:543–550. doi:10.1111/his.12050
50. Fury MG, Sherman E, Ho AL, Xiao H, Tsai F, Nwankwo O, Sima C, Heguy A, Katabi N, Haque S, et al. A phase 1 study of everolimus plus docetaxel plus cisplatin as induction chemotherapy for patients with locally and/or regionally advanced head and neck cancer. Cancer (2013) 119:1823–1831. doi:10.1002/cncr.27986
51. Chang YS, Hsu HT, Ko YC, Yeh KT, Chang SJ, Lin CY, Chang JG. Combined mutational analysis of RAS, BRAF, PIK3CA, and TP53 genes in Taiwanese patients with oral squamous cell carcinoma. Oral Surg Oral Med Oral Pathol Oral Radiol (2014) 118: doi:10.1016/j.oooo.2014.03.016
52. Zhang P, Mirani N, Baisre A, Fernandes H. Molecular heterogeneity of head and neck squamous cell carcinoma defined by next-generation sequencing. Am J Pathol (2014) 184:1323–1330. doi:10.1016/j.ajpath.2014.01.028
53. Lin DC, Meng X, Hazawa M, Nagata Y, Varela AM, Xu L, Sato Y, Liu LZ, Ding LW, Sharma A, et al. The genomic landscape of nasopharyngeal carcinoma. Nat Genet (2014) 46:866–871. doi:10.1038/ng.3006
54. Ross JS, Wang K, Rand J V., Sheehan CE, Jennings TA, Al-Rohil RN, Otto GA, Curran JC, Palmer G, Downing SR, et al. Comprehensive genomic profiling of relapsed and metastatic adenoid cystic carcinomas by next-generation sequencing reveals potential new routes to targeted therapies. Am J Surg Pathol (2014) 38:235–238. doi:10.1097/PAS.0000000000000102
55. Prigge ES, Urban K, Stiegler S, Müller M, Kloor M, Mai S, Ottstadt M, Lohr F, Wenz F, Wagner S, et al. No evidence of oncogenic KRAS mutations in squamous cell carcinomas of the anogenital tract and head and neck region independent of human papillomavirus and p16INK4a status. Hum Pathol (2014) 45:2347–2354. doi:10.1016/j.humpath.2014.08.001
56. Tan DSW, Wang W, Leong HS, Sew PH, Lau DP, Chong FT, Krisna SS, Lim TKH, Iyer NG. Tongue carcinoma infrequently harbor common actionable genetic alterations. BMC Cancer (2014) 14:1–9. doi:10.1186/1471-2407-14-679
57. Chung CH, Lee JW, Slebos RJ, Howard JD, Perez J, Kang H, Fertig EJ, Considine M, Gilbert J, Murphy BA, et al. A 30-UTR KRAS-variant is associated with cisplatin resistance in patients with recurrent and/or metastatic head and neck squamous cell carcinoma. Ann Oncol (2014) 25:2230–2236. doi:10.1093/annonc/mdu367
58. Boeckx C, Weyn C, Vanden Bempt I, Deschoolmeester V, Wouters A, Specenier P, Van Laer C, Van Den Weyngaert D, Kockx M, Vermorken JB, et al. Mutation analysis of genes in the EGFR pathway in Head and Neck cancer patients: Implications for anti-EGFR treatment response. BMC Res Notes (2014) 7: doi:10.1186/1756-0500-7-337
59. Chiosea SI, Miller M, Seethala RR. HRAS Mutations in

Epithelial-Myoepithelial Carcinoma. Head Neck Pathol (2014) 8:146–150. doi:10.1007/s12105-013-0506-4
60. Zhang ZC, Fu S, Wang F, Wang HY, Zeng YX, Shao JY. Oncogene mutational profile in nasopharyngeal carcinoma. Onco Targets Ther (2014) 7:457–467. doi:10.2147/OTT.S58791
61. Franchi A, Innocenti DRD, Palomba A, Miligi L, Paiar F, Franzese C, Santucci M. Low prevalence of K-RAS, EGF-R and BRAF mutations in sinonasal adenocarcinomas. Implications for anti-EGFR treatments. Pathol Oncol Res (2014) 20:571–579. doi:10.1007/s12253-013-9730-1
62. Biswas NK, Das S, Maitra A, Sarin R, Majumder PP. Somatic mutations in arachidonic acid metabolism pathway genes enhance oral cancer post-treatment disease-free survival. Nat Commun (2014) 5:1–9. doi:10.1038/ncomms6835
63. Cortelazzi B, Verderio P, Ciniselli CM, Pizzamiglio S, Bossi P, Gloghini A, Gualeni A V., Volpi CC, Locati L, Pierotti MA, et al. Receptor tyrosine kinase profiles and human papillomavirus status in oropharyngeal squamous cell carcinoma. J Oral Pathol Med (2015) 44:734–745. doi:10.1111/jop.12301
64. Pickering CR, Zhou JH, Lee JJ, Drummond JA, Peng SA, Saade RE, Tsai KY, Curry JL, Tetzlaff MT, Lai SY, et al. Mutational landscape of aggressive cutaneous squamous cell carcinoma. Clin Cancer Res (2014) 20:6582–6592. doi:10.1158/1078-0432.CCR-14-1768
65. Rampias T, Giagini A, Siolos S, Matsuzaki H, Sasaki C, Scorilas A, Psyrri A. RAS/PI3K crosstalk and cetuximab resistance in head and neck squamous cell carcinoma. Clin Cancer Res (2014) 20:2933–2946. doi:10.1158/1078-0432.CCR-13-2721
66. Zhang JW, Qin T, Hong SD, Zhang J, Fang WF, Zhao YY, Yang YP, Xue C, Huang Y, Zhao HY, et al. Multiple oncogenic mutations related to targeted therapy in nasopharyngeal carcinoma. Chin J Cancer (2015) 34: doi:10.1186/s40880-015-0011-0
67. Seiwert TY, Zuo Z, Keck MK, Khattri A, Pedamallu CS, Stricker T, Brown C, Pugh TJ, Stojanov P, Cho J, et al. Integrative and comparative genomic analysis of HPV-positive and HPV-negative head and neck squamous cell carcinomas. Clin Cancer Res (2015) 21:632–641. doi:10.1158/1078-0432.CCR-13-3310
68. Vettore AL, Ramnarayanan K, Poore G, Lim K, Ong CK, Huang KK, Leong HS, Chong FT, Lim TK-H, Lim WK, et al. Mutational landscapes of tongue carcinoma reveal recurrent mutations in genes of therapeutic and prognostic relevance. Genome Med 2015 71 (2015) 7:1–15. doi:10.1186/S13073-015-0219-2
69. Chen SJ, Liu H, Liao CT, Huang PJ, Huang Y, Hsu A, Tang P, Chang YS, Chenx HC, Yen TC. Ultra-deep targeted sequencing of advanced oral squamous cell carcinoma identifies a mutation-based prognostic gene signature. Oncotarget (2015) 6:18066–18080. doi:10.18632/oncotarget.3768
70. Fu Y, Cruz-Monserrate Z, Helen Lin H, Chung Y, Ji B, Lin SM, Vonderfecht S, Logsdon CD, Li CF, Ann DK. Ductal activation of oncogenic KRAS alone induces sarcomatoid phenotype. Sci Rep (2015) 5:1–16. doi:10.1038/srep13347
71. Grünewald I, Vollbrecht C, Meinrath J, Meyer MF, Heukamp LC, Drebber U, Quaas A, Beutner D, Hüttenbrink KB, Wardelmann E, et al. Targeted next generation sequencing of parotid gland cancer uncovers genetic heterogeneity. Oncotarget (2015) 6:18224–18237. doi:10.18632/oncotarget.4015
72. Kato S, Elkin SK, Schwaederle M, Tomson BN, Helsten T, Carter JL, Kurzrock R. Genomic landscape of salivary gland tumors. Oncotarget (2015) 6:25631–25645. doi:10.18632/oncotarget.4554
73. Chiosea SI, Williams L, Griffith CC, Thompson LDR, Weinreb I, Bauman JE, Luvison A, Roy S, Seethala RR, Nikiforova MN. Molecular characterization of apocrine salivary duct carcinoma. Am J Surg Pathol (2015) 39:744–752. doi:10.1097/PAS.0000000000000410
74. Er TK, Wang YY, Chen CC, Herreros-Villanueva M, Liu TC, Yuan SSF. Molecular characterization of oral squamous cell carcinoma using targeted next-generation sequencing. Oral Dis (2015) 21:872–878. doi:10.1111/odi.12357
75. Fonseca I, Bell A, Wani K, Bell D. Global transcriptome and sequenome analysis of formalin-fixed salivary epithelial-myoepithelial carcinoma specimens. Genes Chromosom Cancer (2015) 54:249–259. doi:10.1002/gcc.22238
76. Braig F, Voigtlaender M, Schieferdecker A, Busch CJ, Laban S, Grob T, Kriegs M, Knecht R, Bokemeyer C, Binder M. Liquid biopsy monitoring uncovers acquired RAS-mediated resistance to cetuximab in a substantial proportion of patients with head and neck squamous cell carcinoma. Oncotarget (2016) 7:42988–42995. doi:10.18632/oncotarget.8943
77. Rettig EM, Talbot CC, Sausen M, Jones S, Bishop JA, Wood LD, Tokheim C, Niknafs N, Karchin R, Fertig EJ, et al. Whole-genome sequencing of salivary gland adenoid cystic carcinoma. Cancer Prev Res (2016) 9:265–274. doi:10.1158/1940-6207.CAPR-15-0316
78. Mitani Y, Liu B, Rao PH, Borra VJ, Zafereo M, Weber RS, Kies M, Lozano G, Andrew Futreal P, Caulin C, et al. Novel MYBL1 Gene Rearrangements with Recurrent MYBL1-NFIB Fusions in Salivary Adenoid Cystic Carcinomas Lacking t(6;9) Translocations. Clin Cancer Res (2016) 22:725–733. doi:10.1158/1078-0432.CCR-15-2867-T
79. Drier Y, Cotton MJ, Williamson KE, Gillespie SM, Ryan RJH, Kluk MJ, Carey CD, Rodig SJ, Sholl LM, Afrogheh AH, et al. An oncogenic MYB feedback loop drives alternate cell fates in adenoid cystic carcinoma. Nat Genet (2016) 48:265–272. doi:10.1038/ng.3502
80. Shalmon B, Drendel M, Wolf M, Hirshberg A, Cohen Y. Mutational analysis of PI3K/AKT and RAS/RAF pathway activation in malignant salivary gland tumours with a new mutation of PIK3CA. Int J Oral Maxillofac Surg (2016) 45:721–725. doi:10.1016/j.ijom.2015.12.015
81. Wang K, Russell JS, McDermott JD, Elvin JA, Khaira D, Johnson A, Jennings TA, Ali SM, Murray M, Marshall C, et al. Profiling of 149 salivary duct carcinomas, carcinoma ex pleomorphic adenomas, and adenocarcinomas, not otherwise specified reveals actionable genomic alterations. Clin Cancer Res (2016) 22:6061–6068. doi:10.1158/1078-0432.CCR-15-2568
82. Tinhofer I, Budach V, Saki M, Konschak R, Niehr F, Jöhrens K, Weichert W, Linge A, Lohaus F, Krause M, et al. Targeted next-generation sequencing of locally advanced squamous cell carcinomas of the head and neck reveals druggable targets for improving adjuvant chemoradiation. Eur J Cancer (2016) 57:78–86. doi:10.1016/j.ejca.2016.01.003
83. Schneider T, Strehl A, Linz C, Brands R, Hartmann S, Beckford F, Rosenwald A, Kübler AC, Müller-Richter UDA. Phosphorylated epidermal growth factor receptor expression and KRAS mutation status in salivary gland carcinomas. Clin Oral Investig (2016) 20:541–551. doi:10.1007/s00784-015-1541-1
84. Chiosea SI, Thompson LDR, Weinreb I, Bauman JE, Mahaffey AM, Miller C, Ferris RL, Gooding WE. Subsets of salivary duct carcinoma defined by morphologic evidence of pleomorphic adenoma, PLAG1 or HMGA2 rearrangements, and common genetic alterations. Cancer (2016) 122:3136–3144. doi:10.1002/cncr.30179
85. Dalin MG, Desrichard A, Katabi N, Makarov V, Walsh LA, Lee KW, Wang Q, Armenia J, West L, Dogan S, et al. Comprehensive molecular characterization of salivary duct carcinoma reveals actionable targets and similarity to apocrine breast cancer. Clin Cancer Res (2016) 22:4623–4633. doi:10.1158/1078-0432.CCR-16-0637
86. Wu ES, Park JY, Zeitouni JA, Gomez CR, Reis IM, Zhao W, Kwon D, Lee E, Nelson OL, Lin HY, et al. Effect of actionable somatic mutations on racial/ethnic disparities in head and neck cancer prognosis. Head Neck (2016) 38:1234–1241. doi:10.1002/hed.24420
87. Chau NG, Li YY, Jo VY, Rabinowits G, Lorch JH, Tishler RB, Margalit DN, Schoenfeld JD, Annino DJ, Goguen LA, et al. Incorporation of next-generation sequencing into routine clinical care to direct treatment of head and neck squamous cell carcinoma. Clin Cancer Res (2016) 22:2939–2949. doi:10.1158/1078-0432.CCR-15-2314
88. Bell D, Sniegowski MC, Wani K, Prieto V, Esmaeli B. Mutational landscape of lacrimal gland carcinomas and implications for treatment. Head Neck (2016) 38:E724–E729. doi:10.1002/hed.24078
89. Luk PP, Weston JD, Yu B, Selinger CI, Ekmejian R, Eviston TJ, Lum T, Gao K, Boyer M, O'Toole SA, et al. Salivary duct carcinoma: Clinicopathologic features, morphologic spectrum, and somatic mutations. Head Neck (2016) 38:E1838–E1847. doi:10.1002/hed.24332

90. Al-Hebshi NN, Li S, Nasher AT, El-Setouhy M, Alsanosi R, Blancato J, Loffredo C. Exome sequencing of oral squamous cell carcinoma in users of Arabian snuff reveals novel candidates for driver genes. Int J Cancer (2016) 139:363–372. doi:10.1002/ijc.30068
91. Hedberg ML, Goh G, Chiosea SI, Bauman JE, Freilino ML, Zeng Y, Wang L, Diergaarde BB, Gooding WE, Lui VWY, et al. Genetic landscape of metastatic and recurrent head and neck squamous cell carcinoma. J Clin Invest (2016) 126:169–180. doi:10.1172/JCI82066
92. Feldman R, Gatalica Z, Knezetic J, Reddy S, Nathan CA, Javadi N, Teknos T. Molecular profiling of head and neck squamous cell carcinoma. Head Neck (2016) 38:E1625–E1638. doi:10.1002/hed.24290
93. Ock CY, Son B, Keam B, Lee SY, Moon J, Kwak H, Kim S, Kim TM, Jeon YK, Kwon SK, et al. Identification of genomic mutations associated with clinical outcomes of induction chemotherapy in patients with head and neck squamous cell carcinoma. J Cancer Res Clin Oncol (2016) 142:873–883. doi:10.1007/s00432-015-2083-2
94. Oikawa Y, Morita KI, Kayamori K, Tanimoto K, Sakamoto K, Katoh H, Ishikawa S, Inazawa J, Harada H. Receptor tyrosine kinase amplification is predictive of distant metastasis in patients with oral squamous cell carcinoma. Cancer Sci (2017) 108:256–266. doi:10.1111/cas.13126
95. van Ginkel JH, de Leng WWJ, de Bree R, van Es RJJ, Willems SM. Targeted sequencing reveals TP53 as a potential diagnostic biomarker in the post-treatment surveillance of head and neck cancer. Oncotarget (2016) 7:61575–61586. doi:10.18632/oncotarget.11196
96. Morris LGT, Chandramohan R, West L, Zehir A, Chakravarty D, Pfister DG, Wong RJ, Lee NY, Sherman EJ, Baxi SS, et al. The molecular landscape of recurrent and metastatic head and neck cancers insights from a precision oncology sequencing platform. JAMA Oncol (2017) 3:244–255. doi:10.1001/jamaoncol.2016.1790
97. Zhang L, MacIsaac KD, Zhou T, Huang PY, Xin C, Dobson JR, Yu K. Genomic analysis of nasopharyngeal carcinoma reveals tme-based subtypes. Mol Cancer Res (2017) 15:1722–1732. doi:10.1158/1541-7786.MCR-17-0134
98. Ali SM, Yao M, Yao J, Wang J, Cheng Y, Schrock AB, Chirn GW, Chen H, Mu S, Gay L, et al. Comprehensive genomic profiling of different subtypes of nasopharyngeal carcinoma reveals similarities and differences to guide targeted therapy. Cancer (2017) 123:3628–3637. doi:10.1002/cncr.30781
99. Upadhyay P, Gardi N, Desai S, Chandrani P, Joshi A, Dharavath B, Arora P, Bal M, Nair S, Dutt A. Genomic characterization of tobacco/nut chewing HPV-negative early stage tongue tumors identify MMP10 as a candidate to predict metastases. Oral Oncol (2017) 73:56–64. doi:10.1016/j.oraloncology.2017.08.003
100. Kang H, Tan M, Bishop JA, Jones S, Sausen M, Ha PK, Agrawal N. Whole-exome sequencing of salivary gland mucoepidermoid carcinoma. Clin Cancer Res (2017) 23:283–288. doi:10.1158/1078-0432.CCR-16-0720
101. Dalin MG, Katabi N, Persson M, Lee KW, Makarov V, Desrichard A, Walsh LA, West L, Nadeem Z, Ramaswami D, et al. Multi-dimensional genomic analysis of myoepithelial carcinoma identifies prevalent oncogenic gene fusions. Nat Commun (2017) 8: doi:10.1038/s41467-017-01178-z
102. Li YY, Chung GTY, Lui VWY, To KF, Ma BBY, Chow C, Woo JKS, Yip KY, Seo J, Hui EP, et al. Exome and genome sequencing of nasopharynx cancer identifies NF-? B pathway activating mutations. Nat Commun (2017) 8: doi:10.1038/ncomms14121
103. Dogan S, Chute DJ, Xu B, Ptashkin RN, Chandramohan R, Casanova-Murphy J, Nafa K, Bishop JA, Chiosea SI, Stelow EB, et al. Frequent IDH2 R172 mutations in undifferentiated and poorly-differentiated sinonasal carcinomas. J Pathol (2017) 242:400–408. doi:10.1002/path.4915
104. Zehir A, Benayed R, Shah RH, Syed A, Middha S, Kim HR, Srinivasan P, Gao J, Chakravarty D, Devlin SM, et al. Mutational landscape of metastatic cancer revealed from prospective clinical sequencing of 10,000 patients. Nat Med (2017) 23:703–713. doi:10.1038/nm.4333
105. Bersani C, Sivars L, Haeggblom L, DiLorenzo S, Mints M, ährlund-Richter A, Tertipis N, Munck-Wikland E, Näsman A, Ramqvist T, et al. Targeted sequencing of tonsillar and base of tongue cancer and human papillomavirus positive unknown primary of the head and neck reveals prognostic effects of mutated FGFR3. Oncotarget (2017) 8:35339–35350. doi:10.18632/oncotarget.15240
106. Nakagaki T, Tamura M, Kobashi K, Koyama R, Fukushima H, Ohashi T, Idogawa M, Ogi K, Hiratsuka H, Tokino T, et al. Profiling cancer-related gene mutations in oral squamous cell carcinoma from Japanese patients by targeted amplicon sequencing. Oncotarget (2017) 8:59113–59122. doi:10.18632/oncotarget.19262
107. Su SC, Lin CW, Liu YF, Fan WL, Chen MK, Yu CP, Yang WE, Su CW, Chuang CY, Li WH, et al. Exome sequencing of oral squamous cell carcinoma reveals molecular subgroups and novel therapeutic opportunities. Theranostics (2017) 7:1088–1099. doi:10.7150/thno.18551
108. Robinson DR, Wu YM, Lonigro RJ, Vats P, Cobain E, Everett J, Cao X, Rabban E, Kumar-Sinha C, Raymond V, et al. Integrative clinical genomics of metastatic cancer. Nature (2017) 548:297–303. doi:10.1038/nature23306
109. Khoo TK, Yu B, Smith JA, Clarke AJ, Luk PP, Selinger CI, Mahon KL, Kraitsek S, Palme C, Boyer MJ, et al. Somatic mutations in salivary duct carcinoma and potential therapeutic targets. Oncotarget (2017) 8:75893–75903. doi:10.18632/oncotarget.18173
110. Saida K, Murase T, Ito M, Fujii K, Takino H, Masaki A, Kawakita D, Ijichi K, Tada Y, Kusafuka K, et al. Mutation analysis of the EGFR pathway genes, EGFR, RAS, PIK3CA, BRAF, and AKT1, in salivary gland adenoid cystic carcinoma. Oncotarget (2018) 9:17043–17055. doi:10.18632/oncotarget.24818
111. El Hallani S, Udager AM, Bell D, Fonseca I, Thompson LDR, Assaad A, Agaimy A, Luvison AM, Miller C, Seethala RR, et al. Epithelial-Myoepithelial Carcinoma: Frequent Morphologic and Molecular Evidence of Preexisting Pleomorphic Adenoma, Common HRAS Mutations in PLAG1-intact and HMGA2-intact Cases, and Occasional TP53, FBXW7, and SMARCB1 Alterations in High-grade Cases. Am J Surg Pathol (2018) 42:18–27. doi:10.1097/PAS.0000000000000933
112. Shimura T, Tada Y, Hirai H, Kawakita D, Kano S, Tsukahara K, Shimizu A, Takase S, Imanishi Y, Ozawa H, et al. Prognostic and histogenetic roles of gene alteration and the expression of key potentially actionable targets in salivary duct carcinomas. Oncotarget (2018) 9:1852–1867. doi:10.18632/oncotarget.22927
113. Perdomo S, Anantharaman D, Foll M, Abedi-Ardekani B, Durand G, Reis Rosa LA, Holmila R, Le Calvez-Kelm F, Tajara EH, Wünsch-Filho V, et al. Genomic analysis of head and neck cancer cases from two high incidence regions. PLoS One (2018) 13: doi:10.1371/journal.pone.0191701
114. Vossen DM, Verhagen CVM, Verheij M, Wessels LFA, Vens C, van den Brekel MWM. Comparative genomic analysis of oral versus laryngeal and pharyngeal cancer. Oral Oncol (2018) 81:35–44. doi:10.1016/j.oraloncology.2018.04.006
115. Dubot C, Bernard V, Sablin MP, Vacher S, Chemlali W, Schnitzler A, Pierron G, Ait Rais K, Bessoltane N, Jeannot E, et al. Comprehensive genomic profiling of head and neck squamous cell carcinoma reveals FGFR1 amplifications and tumour genomic alterations burden as prognostic biomarkers of survival. Eur J Cancer (2018) 91:47–55. doi:10.1016/j.ejca.2017.12.016
116. Mirghani H, Lacroix L, Rossoni C, Sun R, Aupérin A, Casiraghi O, Villepelet A, Lacave R, Faucher G, Marty V, et al. Does smoking alter the mutation profile of human papillomavirus–driven head and neck cancers? Eur J Cancer (2018) 94:61–69. doi:10.1016/j.ejca.2018.02.013
117. Nakagaki T, Tamura M, Kobashi K, Omori A, Koyama R, Idogawa M, Ogi K, Hiratsuka H, Tokino T, Sasaki Y. Targeted next-generation sequencing of 50 cancer-related genes in Japanese patients with oral squamous cell carcinoma. Tumor Biol (2018) 40: doi:10.1177/1010428318800180
118. Li X, Zhao Z, Yi S, Ma L, Li M, Liu M, Zhang Y, Liu G. Nuclear Klf4 accumulation is associated with cetuximab drug-resistance and predicts poor prognosis of nasopharyngeal carcinoma. J Transl Med (2018) 16: doi:10.1186/s12967-018-1561-0
119. Batta N, Pandey M. Mutational spectrum of tobacco associated oral squamous carcinoma and its therapeutic

significance. World J Surg Oncol (2019) 17:1–12. doi:10.1186/s12957-019-1741-2
120. Akagi Y, Tachibana T, Orita Y, Gion Y, Marunaka H, Makino T, Miki K, Akisada N, Yoshino T, Nishizaki K, et al. KRAS mutations in tongue squamous cell carcinoma. Acta Otolaryngol (2019) 139:647–651. doi:10.1080/00016489.2019.1610574
121. Chung AK, OuYang CN, Liu H, Chao M, Luo JD, Lee CY, Lu YJ, Chung IC, Chen LC, Wu SM, et al. Targeted sequencing of cancer-related genes in nasopharyngeal carcinoma identifies mutations in the TGF-β pathway. Cancer Med (2019) 8:5116–5127. doi:10.1002/cam4.2429
122. Reder H, Wagner S, Gamerdinger U, Sandmann S, Wuerdemann N, Braeuninger A, Dugas M, Gattenloehner S, Klussmann JP, Wittekindt C. Genetic alterations in human papillomavirus-associated oropharyngeal squamous cell carcinoma of patients with treatment failure. Oral Oncol (2019) 93:59–65. doi:10.1016/j.oraloncology.2019.04.013
123. Stanek L, Glendova K, Tesarova P, Gurlich R, Holeckova P, Musil Z, Hrudka J, Pala M, Mateicka F, Chovanec M. Molecular and IHC analysis of head and neck carcinomas associated with HPV infection. Bratislava Med J (2019) 120:832–838. doi:10.4149/BLL_2019_138
124. Nakaguro M, Urano M, Ogawa I, Hirai H, Yamamoto Y, Yamaguchi H, Tanigawa M, Matsubayashi J, Hirano H, Shibahara J, et al. Histopathological evaluation of minor salivary gland papillary–cystic tumours: focus on genetic alterations in sialadenoma papilliferum and intraductal papillary mucinous neoplasm. Histopathology (2020) 76:411–422. doi:10.1111/his.13990
125. Wang H, Li H, Hu L, Zhou J, Zhai C, Wang D, Sun X. EGFR and KRAS mutations in Chinese patients with sinonasal inverted papilloma and oncocytic papilloma. Histopathology (2019) 75:274–281. doi:10.1111/his.13868
126. Haft S, Ren S, Xu G, Mark A, Fisch K, Guo TW, Khan Z, Pang J, Ando M, Liu C, et al. Mutation of chromatin regulators and focal hotspot alterations characterize human papillomavirus–positive oropharyngeal squamous cell carcinoma. Cancer (2019) 125:2423–2434. doi:10.1002/cncr.32068
127. Smith JD, Birkeland AC, Rosko AJ, Hoesli RC, Foltin SK, Swiecicki P, Mierzwa M, Chinn SB, Shuman AG, Malloy KM, et al. Mutational profiles of persistent/recurrent laryngeal squamous cell carcinoma. Head Neck (2019) 41:423–428. doi:10.1002/hed.25444
128. Westbrook TC, Hagemann IS, Ley J, Chen K, Palka K, Liu J, Chen L, Oppelt P, Adkins D. Prospective assessment of the clinical benefit of a tailored cancer gene set built on a next-generation sequencing platform in patients with recurrent or metastatic head and neck cancer. Med Oncol (2020) 37:1–8. doi:10.1007/s12032-019-1336-3
129. Samstein RM, Lee CH, Shoushtari AN, Hellmann MD, Shen R, Janjigian YY, Barron DA, Zehir A, Jordan EJ, Omuro A, et al. Tumor mutational load predicts survival after immunotherapy across multiple cancer types. Nat Genet (2019) 51:202–206. doi:10.1038/s41588-018-0312-8
130. Priestley P, Baber J, Lolkema MP, Steeghs N, de Bruijn E, Shale C, Duyvesteyn K, Haidari S, van Hoeck A, Onstenk W, et al. Pan-cancer whole-genome analyses of metastatic solid tumours. Nature (2019) 575:210–216. doi:10.1038/s41586-019-1689-y
131. Sayáns MP, Petronacci CMC, Pouso AIL, Iruegas EP, Carrión AB, Peñaranda JMS, García AG. Comprehensive genomic review of TCGA head and neck squamous cell carcinomas (HNSCC). J Clin Med (2019) 8:1896. doi:10.3390/jcm8111896
132. Kobayashi K, Yoshimoto S, Matsumoto F, Ando M, Murakami N, Omura G, Fukasawa M, Matsumoto Y, Matsumura S, Akamatsu M, et al. All-Exon TP53 Sequencing and Protein Phenotype Analysis Accurately Predict Clinical Outcome after Surgical Treatment of Head and Neck Squamous Cell Carcinoma. Ann Surg Oncol (2019) 26: doi:10.1245/s10434-019-07287-x
133. Dogan S, Ng CKY, Xu B, Kumar R, Wang L, Edelweiss M, Scott SN, Zehir A, Drilon A, Morris LGT, et al. The repertoire of genetic alterations in salivary duct carcinoma including a novel HNRNPH3-ALK rearrangement. Hum Pathol (2019) 88:66–77. doi:10.1016/j.humpath.2019.03.004
134. Gauthaman A, Moorthy A. Prevalence of K-ras Codon 12 Mutations in Indian Patients with Head and Neck Cancer. Indian J Clin Biochem (2021) 36:370–374. doi:10.1007/s12291-020-00882-w
135. Kyurkchiyan SG, Popov TM, Kachakova D, Mihova K, Petkova VY, Rangachev J, Mitev VI, Popova DP, Kaneva RP. Pathogenic somatic alterations in advanced hpv-negative cell squamous laryngeal carcinoma revealed via targeted next generation sequencing. Genetika (2020) 52:477–494. doi:10.2298/GENSR2002477K
136. Morita M, Murase T, Okumura Y, Ueda K, Sakamoto Y, Masaki A, Kawakita D, Tada Y, Nibu KI, Shibuya Y, et al. Clinicopathological significance of EGFR pathway gene mutations and CRTC1/3–MAML2 fusions in salivary gland mucoepidermoid carcinoma. Histopathology (2020) 76:1013–1022. doi:10.1111/his.14100
137. Morfouace M, Stevovic A, Vinches M, Golfinopoulos V, Jin DX, Holmes O, Erlich R, Fayette J, Croce S, Ray-Coquard I, et al. First results of the EORTC-SPECTA/Arcagen study exploring the genomics of rare cancers in collaboration with the European reference network EURACAN. ESMO Open (2020) 5: doi:10.1136/esmoopen-2020-001075
138. Kawamura K, Daa T, Kawano K, Yokoyama S. Activation of the RAS/ERK signaling pathway by RASAL1 and its clinical significance in squamous cell carcinomas of the tongue. J Oral Maxillofac Surgery, Med Pathol (2020) 32:400–405. doi:10.1016/j.ajoms.2020.01.002
139. Jayaprakash C, Varghese VK, Jayaram P, Chakrabarty S, Kudva A, Ray S, Satyamoorthy K. Relevance and actionable mutational spectrum in oral squamous cell carcinoma. J Oral Pathol Med (2020) 49:427–434. doi:10.1111/jop.12985
140. Leblanc O, Vacher S, Lecerf C, Jeannot E, Klijanienko J, Berger F, Hoffmann C, Calugaru V, Badois N, Chilles A, et al. Biomarkers of cetuximab resistance in patients with head and neck squamous cell carcinoma. Cancer Biol Med (2020) 17:208–217. doi:10.20892/j.issn.2095-3941.2019.0153
141. Mueller SA, Gauthier MEA, Blackburn J, Grady JP, Kraitsek S, Hajdu E, Dettmer MS, Dahlstrom JE, Lee CS, Luk PP, et al. Molecular patterns in salivary duct carcinoma identify prognostic subgroups. Mod Pathol (2020) 33:1896–1909. doi:10.1038/s41379-020-0576-2
142. Kim Y, Song S, Lee M, Swatloski T, Kang JH, Ko YH, Park WY, Jeong HS, Park K. Integrative genomic analysis of salivary duct carcinoma. Sci Rep (2020) 10: doi:10.1038/s41598-020-72096-2
143. Hsieh MS, Lee YH, Jin YT, Kuo YJ. Clinicopathological study of intraductal carcinoma of the salivary gland, with emphasis on the apocrine type. Virchows Arch (2020) 477:581–592. doi:10.1007/s00428-020-02823-7
144. ORCA-IN | ICGC Data Portal. Available at: https://dcc.icgc.org/genes/ENSG00000134982/mutations%0Ahttps://dcc.icgc.org/projects/ORCA-IN [Accessed September 13, 2021]
145. Nakaguro M, Tanigawa M, Hirai H, Yamamoto Y, Urano M, Takahashi RH, Sukeda A, Okumura Y, Honda S, Tasaki K, et al. The Diagnostic Utility of RAS Q61R Mutation-specific Immunohistochemistry in Epithelial-Myoepithelial Carcinoma. Am J Surg Pathol (2021) 45:885–894. doi:10.1097/PAS.0000000000001673
146. Sánchez-Fernández P, Riobello C, Costales M, Vivanco B, Cabal VN, García-Marín R, Suárez-Fernández L, López F, Cabanillas R, Hermsen MA, et al. Next-generation sequencing for identification of actionable gene mutations in intestinal-type sinonasal adenocarcinoma. Sci Rep (2021) 11:2247. doi:10.1038/s41598-020-80242-z
147. Consortium TAPG. AACR Project GENIE: Powering Precision Medicine through an International Consortium. Cancer Discov (2017) 7:818–831. doi:10.1158/2159-8290.CD-17-0151
148. Reder H, Wagner S, Wuerdemann N, Langer C, Sandmann S, Braeuninger A, Dugas M, Gattenloehner S, Wittekindt C, Klussmann JP. Mutation patterns in recurrent and/or metastatic oropharyngeal squamous cell carcinomas in relation to human papillomavirus status. Cancer Med (2021) 10:1347–1356. doi:10.1002/cam4.3741
149. Patel K, Bhat FA, Patil S, Routray S, Mohanty N, Nair B, Sidransky D, Ganesh MS, Ray JG, Gowda H, et al. Whole-Exome Sequencing Analysis of Oral Squamous Cell Carcinoma Delineated by Tobacco Usage Habits. Front Oncol (2021) 11: doi:10.3389/fonc.2021.660696

**Table S2: Detailed characteristics of the studies included in the meta-analysis**

| | HRAS | | | KRAS | | | NRAS | | | HPV status | Sampling technique | Risk factors | Geographical region |
|---|---|---|---|---|---|---|---|---|---|---|---|---|---|
| Cohort | cases | cohort size | % | cases | cohort size | % | cases | cohort size | % | | | | |
| Das et al., 2000 | 14 | 50 | 28.00 | 4 | 12 | 33.33 | 0 | 50 | 0.00 | NP | PCR+Direct seq | T, A, B | India |
| Yoo et al., 2000 | 5 | 24 | 20.83 | 2 | 24 | 8.33 | | | | NP | PCR+Direct seq | NP | USA |
| Yoo et al., 2000 | 9 | 50 | 18.00 | | | | | | | NP | PCR+Direct seq | NP | USA |
| Lin et al., 2002 | 10 | 28 | 35.71 | | | | | | | NP | PCR+Direct seq | NP | China |
| Weber et al., 2003 | | | | 5 | 89 | 5.62 | | | | NP | PCR+Direct seq | NP | Germany |
| Perrone et al., 2003 | 0 | 18 | 0.00 | | | | | | | NP | PCR+Direct seq | W | Italy |
| Ruiz-Godoy et al., 2006 | | | | 0 | 20 | 0.00 | | | | NP | PCR+Direct seq | A, S | Mexico |
| Sathyan et al., 2006 | | | | | | | | | | NP | PCR-SSCP | NP | India |
| Frattini et al., 2006 | | | | 9 | 18 | 50.00 | | | | NP | PCR+Direct seq | W | Italy |
| Augello et al., 2006 | 11 | 33 | 33.33 | 2 | 33 | 6.06 | | | | NP | SSCP | NP | Italy |
| Russo et al., 2006 | 5 | 81 | 6.17 | 0 | 81 | 0.00 | 0 | 81 | 0.00 | NP | PCR+Direct seq | NP | Italy |
| Sathyan et al., 2007 | 19 | 152 | 12.50 | 0 | 152 | 0.00 | 0 | 152 | 0.00 | NP | PCR+Direct seq | T, A | India |
| Gupta et al., 2007 | | | | 0 | 35 | 0.00 | | | | NP | PCR+SSCP | NP | India |
| Sheikh Ali et al., 2008 | | | | 0 | 91 | 0.00 | | | | NP | PCR+Direct seq | NP | Japan |
| Chou et al., 2008 | | | | 0 | 45 | 0.00 | | | | NP | PCR+Direct seq | NP | Taiwan |
| Bornholdt et al., 2008 | | | | 8 | 174 | 4.60 | | | | NP | PCR+Direct seq | W, S, C | Denmark |

| | | | | | | | | | | | | | |
|---|---|---|---|---|---|---|---|---|---|---|---|---|---|
| Murugan et al., 2008 | 3 | 4 | 75.00 | | | | | | | HPV Neg | PCR+Direct seq | NP | India |
| Dahse et al., 2009 | | | | 1 | 65 | 1.54 | | | | NP | PCR+Direct seq | NP | Germany |
| Murugan et al., 2009 | 10 | 56 | 17.86 | | | | | | | NP | PCR+Direct seq | T, A | Vietnam |
| Bruckman et al., 2010 | | | | 1 | 42 | 2.38 | | | | NP | PCR+Direct seq | NP | USA |
| van Damme et al., 2010 | | | | 1 | 22 | 4.55 | | | | NP | PCR+Direct seq | NP | Belgium |
| Chang et al., 2010 | 8 | 58 | 13.79 | 0 | 58 | 0.00 | 0 | 58 | 0.00 | NP | PCR+Direct seq | NP | Taiwan |
| Tetsu et al., 2010 | 8 | 17 | 47.06 | 1 | 17 | 5.88 | 1 | 17 | 5.88 | NP | PCR+Direct seq | NP | USA |
| Murray et al., 2010 | | | | 1 | 92 | 1.09 | | | | NP | PCR+Direct seq | T, A | Greece |
| Popovic et al., 2010 | 13 | 60 | 21.67 | | | | | | | NP | PCR+Direct seq | S | Serbia |
| Agarwal et al., 2011 | 5 | 120 | 4.17 | 0 | 120 | 0.00 | 0 | 120 | 0.00 | Both | WES | T, A | USA |
| Stransky et al., 2011 | 4 | 74 | 5.41 | 1 | 74 | 1.35 | 0 | 74 | 0.00 | Both | WES | T, A | USA |
| Cohen et al., 2011 | 0 | 37 | 0.00 | 0 | 37 | 0.00 | 0 | 37 | 0.00 | NP | MASS SPEC and direct seq | NP | Israel |
| Wang et al., 2011 | | | | 0 | 47 | 0.00 | | | | NP | PCR+Direct seq | NP | Taiwan |
| Friedland et al., 2011 | | | | 0 | 60 | 0.00 | | | | Both | PCR+SSCP | S, A | Australia |
| Szabo et al., 2011 | | | | 2 | 71 | 2.82 | | | | Both | PCR+ RFLP | S, A | Hungary |
| Suda et al., 2012 | | | | 3 | 115 | 2.61 | | | | NP | PCR+Direct seq | T | Japan |
| Smilek et al. 2012 | | | | 4 | 28 | 14.29 | | | | NP | qPCR | NP | Czech republic |
| Koumaki et al., 2012 | 6 | 86 | 6.98 | | | | | | | NP | PCR+Direct seq | NP | Greece |
| Lopez et al., 2012 | | | | 7 | 58 | 12.07 | | | | NP | PCR+Direct seq | W, T | Spain |
| Bissada et al., 2013 | | | | 7 | 195 | 3.59 | | | | NP | PCR+ RFLP | NP | Canada |

| | | | | | | | | | | | | | |
|---|---|---|---|---|---|---|---|---|---|---|---|---|---|
| Choisea et al., 2013 | 1 | 58 | 1.72 | | | | | | | HPV Pos | PCR+Direct seq | HPV, S | USA |
| Lechner et al., 2013 | 0 | 34 | 0.00 | 2 | 34 | 5.88 | 0 | 34 | 0.00 | Both | MassArray platform | S, A | UK |
| Pickering et al., 2013 | 5 | 40 | 12.50 | 0 | 40 | 0.00 | 0 | 40 | 0.00 | NP | WES | S, A | USA |
| Ho et al., 2013 | 5 | 59 | 8.47 | 0 | 59 | 0.00 | 1 | 59 | 1.69 | NP | WES | NP | USA |
| Stephens et al., 2013 | 0 | 24 | 0.00 | 0 | 24 | 0.00 | 0 | 24 | 0.00 | NP | WES | NP | UK |
| Liu et al., 2013 | 7 | 151 | 4.64 | 2 | 151 | 1.32 | 0 | 151 | 0.00 | Both | WES | NP | USA |
| Cros et al., 2013 | 7 | 107 | 6.54 | 4 | 107 | 3.74 | 2 | 107 | 1.87 | NP | PCR+Direct seq | NP | France |
| Fuji et al., 2013 | | | | 0 | 183 | 0.00 | | | | NP | PCR+Direct seq | S, A | Japan |
| Projetti et al., 2013 | | | | 2 | 34 | 5.88 | | | | NP | High Resolution Melting Analysis | W | France |
| Szablewski et al., 2013 | | | | 12 | 28 | 42.86 | | | | NP | High Resolution Melting Analysis | W, S | France |
| Carvalho et al., 2013 | | | | 0 | 94 | 0.00 | | | | NP | PCR+Direct seq | S, A | Brazil |
| Zanaruddin et al., 2013 | 3 | 107 | 2.80 | 0 | 107 | 0.00 | 0 | 107 | 0.00 | Both | MassArray platform | S, A, B | Malaysia |
| Wetterskog et al., 2013 | 1 | 48 | 2.08 | 0 | 48 | 0.00 | 0 | 48 | 0.00 | NP | PCR+Direct seq | NP | USA |
| Fury et al., 2013 | | | | 1 | 18 | 5.56 | 0 | 18 | 0.00 | Both | MASS SPEC and direct seq | S | USA |
| Chang et al., 2014 | 10 | 79 | 12.66 | 0 | 79 | 0.00 | 0 | 79 | 0.00 | NP | Primer extension analysis | S, A, B | Taiwan |
| Zhang et al., 2014 | 1 | 53 | 1.89 | 6 | 53 | 11.32 | 6 | 53 | 11.32 | Both | Targeted NGS | S, A | USA |
| Lin et al., 2014 | 0 | 56 | 0.00 | 2 | 122 | 1.79 | 1 | 122 | 1.79 | NP | Targeted NGS WES, SNP array | EBV | Singapore |
| Ross et al., 2014 | 0 | 15 | 0.00 | 0 | 15 | 0.00 | 0 | 15 | 0.00 | NP | Targeted NGS | NP | USA |
| Prigge et al., 2014 | | | | 0 | 25 | 0.00 | | | | HPV Positiv e | PCR+Direct seq | NP | Germany |
| Tan et al., 2014 | | | | 0 | 66 | 0.00 | | | | NP | MassArray platform | S | Singapore |

| | | | | | | | | | | | | | |
|---|---|---|---|---|---|---|---|---|---|---|---|---|---|
| Chung et al., 2014 | | | | | | | | | | Both | Micro Array | NP | USA |
| Boeckx et al., 2014 | | | | 1 | 43 | 2.33 | | | | Both | High Resolution Melting Analysis and KRASstrip Assay | S | Belgium |
| Choisea et al., 2014 | 4 | 14 | 28.57 | | | | | | | NP | PCR+Direct seq | NP | USA |
| Zhang et al., 2014 | 1 | 123 | 0.81 | 0 | 123 | 0.00 | 5 | 123 | 4.07 | NP | MassArray platform | NP | china |
| Franchi et al., 2014 | | | | 1 | 27 | 3.70 | | | | NP | PCR+Direct seq | W | Italy |
| Biswas et al., 2014 | 7 | 84 | 8.33 | | | | | | | Both | WES | S, A, B | India |
| Cortelazzi et al., 2014 | 0 | 54 | 0.00 | | | | | | | Both | PCR+Direct seq | NP | Italy |
| Pickering et al., 2014 | 8 | 39 | 20.51 | 0 | 39 | 0.00 | 2 | 39 | 5.13 | NP | WES | NP | USA |
| Rampias et al., 2014 | 17 | 180 | 9.44 | | | | | | | NP | PCR+Direct seq | S, A | Greece |
| Zhang et al., 2015 | | | | 1 | 70 | 1.43 | | | | NP | SnapShot Multiplex assay | S, EBV | china |
| Seiwert et al., 2015 | 4 | 120 | 3.33 | 4 | 120 | 3.33 | 1 | 120 | 0.83 | Both | Targeted NGS | S, A | USA |
| Vettore et al., 2015 | 1 | 60 | 1.67 | | | | | | | NP | Targeted NGS | T | Singapore |
| Chen et al., 2015 | 32 | 345 | 9.28 | 8 | 345 | 2.32 | 4 | 345 | 1.15 | NP | Targeted NGS | S, A, B | Taiwan |
| Fu et al., 2015 | | | | 2 | 18 | 11.11 | 0 | 18 | 0.00 | NP | MassArray platform | NP | Taiwan |
| Grunewald et al., 2015 | 20 | 84 | 23.81 | 0 | 84 | 0.00 | 2 | 84 | 2.38 | NP | Targeted NGS | NP | Germany |
| Kato et al., 2015 | 13 | 117 | 11.11 | 4 | 117 | 3.42 | | | | NP | Targeted NGS | NP | USA |
| Choisea et al., 2015 | 10 | 29 | 34.48 | 0 | 29 | 0.00 | 0 | 29 | 0.00 | NP | Targeted NGS | NP | USA |
| Er et al., 2015 | | | | 0 | 50 | 0.00 | 0 | 50 | | NP | Targeted NGS | S, A, B | Taiwan |
| Fonseca et al., 2015 | 0 | 17 | 0.00 | 3 | 17 | 17.65 | 1 | 17 | 5.88 | NP | Targeted NGS | NP | USA |
| Braig et al., 2016 | 5 | 46 | 10.87 | 2 | 46 | 4.35 | 2 | 46 | 4.35 | Both | Targeted NGS | NP | Germany |

| | | | | | | | | | | | | | |
|---|---|---|---|---|---|---|---|---|---|---|---|---|---|
| Rettig et al., 2016 | 0 | 25 | 0.00 | 0 | 25 | 0.00 | 1 | 25 | 4.00 | NP | WGS | NP | USA |
| Mitani et al., 2016 | 0 | 65 | 0.00 | 0 | 65 | 0.00 | 0 | 65 | 0.00 | NP | WGS | NP | USA |
| Drier et al., 2016 | 0 | 10 | 0.00 | 0 | 19 | 0.00 | 0 | 10 | 0.00 | NP | WGS | NP | USA |
| Shalmon et al., 2016 | | | | 0 | 21 | 0.00 | | | | NP | PCR+Direct seq | NP | Israel |
| Wang et al., 2016 | 21 | 149 | 14.09 | 0 | 149 | 0.00 | 0 | 149 | 0.00 | NP | Targeted NGS | NP | USA |
| Tinhofer et al., 2016 | 1 | 179 | 0.56 | 5 | 179 | 2.79 | 2 | 179 | 1.12 | HPV Pos | Targeted NGS | S | Germany |
| Schneider et al., 2016 | | | | 0 | 43 | 0.00 | | | | NP | PCR+Direct seq | NP | Germany |
| Choisea et al., 2016 | 13 | 38 | 34.21 | | | | | | | NP | Targeted NGS | NP | USA |
| Dalin et al., 2016 | 7 | 31 | 22.58 | 0 | 31 | 0.00 | 0 | 31 | 0.00 | NP | WES | NP | USA |
| Wu et al., 2016 | 8 | 214 | 3.74 | 18 | 214 | 8.41 | | | | NP | MassArray platform | NP | USA |
| Chau et al., 2016 | 10 | 213 | 4.69 | 1 | 213 | 0.47 | 2 | 213 | 0.94 | NP | Targeted NGS | NP | USA |
| Bell et al., 2016 | 0 | 24 | 0.00 | 10 | 24 | 41.67 | 2 | 24 | 8.33 | NP | MassArray platform | NP | USA |
| Luk et al., 2016 | 3 | 23 | 13.04 | 0 | 23 | 0.00 | 1 | 23 | 4.35 | NP | MassArray platform | NP | Australia |
| Al-Hebshi et al., 2016 | 3 | 20 | 15.00 | 0 | 20 | 0.00 | 0 | 20 | 0.00 | HPV Neg | WES | S, EBV | Yemen |
| Hedberg et al., 2016 | 0 | 12 | 0.00 | 1 | 12 | 8.33 | 1 | 12 | 8.33 | NP | WES | NP | USA |
| Feldman et al., 2016 | 8 | 299 | 2.68 | 11 | 448 | 2.46 | 4 | 381 | 1.05 | Both | sanger and Targeted NGS | NP | USA |
| Ock et al., 2016 | 26 | 71 | 36.62 | 13 | 71 | 18.31 | 20 | 71 | 28.17 | NP | Targeted NGS | S | Korea |
| Oikawa et al.,2016 | 4 | 220 | 1.82 | 1 | 220 | 0.45 | 2 | 220 | 0.91 | NP | Targeted NGS | NP | Japan |
| Ginkel et al., 2016 | 10 | 110 | 9.09 | 2 | 110 | 1.82 | 0 | 110 | 0.00 | Both | Targeted NGS | S, A | Netherlands |
| Morris et al., 2017 | 9 | 151 | 5.96 | 2 | 151 | 1.32 | 2 | 151 | 1.32 | Both | Targeted NGS | S, A | USA |
| Zhang et al., 2017 | 2 | 94 | 2.13 | 3 | 94 | 3.19 | 1 | 94 | 1.06 | EBV pos | WES | S, EBV | China |

| | | | | | | | | | | | | | |
|---|---|---|---|---|---|---|---|---|---|---|---|---|---|
| Ali et al., 2017 | 1 | 190 | 0.53 | 3 | 190 | 1.58 | 4 | 190 | 2.11 | Not provided | Targeted NGS | EBV | China |
| Upadhyay et al., 2017 | 3 | 25 | 12.00 | 0 | 25 | 0.00 | 0 | 25 | 0.00 | NP | WES | S, B | India |
| Kang et al., 2017 | 1 | 18 | 5.56 | 0 | 18 | 0.00 | 0 | 18 | 0.00 | NP | WES | T | USA |
| Dalin et al., 2017 | 1 | 40 | 2.50 | 0 | 40 | 0.00 | 0 | 40 | 0.00 | NP | WES | NP | USA |
| Li et al., 2017 | 2 | 105 | 1.90 | 1 | 105 | 0.95 | 4 | 105 | 3.81 | NP | WES | NP | USA/Hong Kong |
| Dogan et al., 2017 | 0 | 30 | 0.00 | 3 | 40 | 7.50 | 0 | 30 | 0.00 | NP | Targeted NGS | NP | USA |
| Zehir et al., 2017 | 11 | 283 | 10.48 | 3 | 283 | 2.86 | 4 | 283 | 3.81 | NP | Targeted NGS | NP | USA |
| Bersani et al., 2017 | 5 | 344 | 1.45 | 9 | 344 | 2.62 | 4 | 344 | 1.16 | Both | Targeted NGS | S | Sweden |
| Nakagaki et al., 2017 | 2 | 47 | 4.26 | 0 | 47 | 0.00 | 0 | 47 | 0.00 | NP | Targeted NGS | NP | Japan |
| Su et al., 2017 | 14 | 120 | 11.67 | | | | | | | NP | WES | S, A, B | Taiwan |
| Robinson et al., 2017 | 3 | 29 | 20.00 | 1 | 29 | 6.67 | 0 | 29 | 0.00 | NP | WES | NP | USA |
| Khoo et al., 2017 | 4 | 15 | 26.67 | 4 | 14 | 28.57 | 4 | 15 | 26.67 | NP | Targeted NGS | NP | Australia |
| Saida et al., 2018 | 4 | 70 | 5.71 | 6 | 70 | 8.57 | 0 | 70 | 0.00 | NP | Micro Array | NP | Japan |
| Hallani et al., 2018 | 8 | 23 | 34.78 | 0 | 23 | 0.00 | 0 | 23 | 0.00 | NP | Targeted NGS | NP | USA |
| Shimura et al., 2018 | 23 | 140 | 16.43 | 0 | 140 | 0.00 | 0 | 140 | 0.00 | NP | PCR+Direct seq | NP | Multi centered |
| Perdomo et al., 2018 | 20 | 180 | 11.11 | | | | | | | Both | Targeted NGS | S, A, HPV | Europe and South America |
| Vossen etal., 2018 | 2 | 111 | 1.80 | 0 | 111 | 0.00 | 1 | 111 | 0.90 | HPV neg | Targeted NGS | S, A | Netherlands |
| Dubot et al.,2018 | 2 | 122 | 1.64 | 2 | 122 | 1.64 | 1 | 122 | 0.82 | Both | PCR+Direct seq | S, A, HPV | France |
| Mirghani et al., 2018 | 0 | 62 | 0.00 | 2 | 62 | 3.23 | 0 | 62 | 0.00 | HPV Pos | Targeted NGS | S, T, HPV | France |

| | | | | | | | | | | | | | |
|---|---|---|---|---|---|---|---|---|---|---|---|---|---|
| Nakagaki et al., 2018 | 4 | 80 | 5.00 | 0 | 80 | 0.00 | 0 | 80 | 0.00 | Both | Targeted NGS | NP | Japan |
| Li et al., 2018 | 34 | 168 | 20.24 | | | | | | | NP | Amplification-refractory mutation system | NP | China |
| Batta et al., 2019 | 8 | 46 | 17.39 | 0 | 46 | 0.00 | 0 | 46 | 0.00 | NP | Targeted NGS | T | India |
| Akagi et al., 2019 | | | | 1 | 85 | 1.18 | | | | NP | PCR+Direct seq | NP | Japan |
| Chung et al., 2019 | 0 | 33 | 0.00 | 1 | 33 | 3.03 | 0 | 33 | 0.00 | NP | Targeted NGS | S, EBV | Taiwan |
| Reder et al., 2019 | 5 | 24 | 41.67 | 1 | 24 | 8.33 | 1 | 24 | 8.33 | NP | Targeted NGS | S, A | Germany |
| Stanek et al., 2019 | | | | 7 | 55 | 12.73 | 2 | 54 | 3.70 | Both | PCR- mutation detection kit | NP | Czech republic |
| Nakaguro et al., 2019 | 7 | 21 | 33.33 | | | | | | | NP | PCR+Direct seq | NP | Japan |
| Wang et al., 2019 | | | | 30 | 80 | 37.50 | | | | NP | PCR+Direct seq | S | China |
| Haft et al., 2019 | 2 | 46 | 4.35 | 0 | 46 | 0.00 | 0 | 46 | 0.00 | HPV Pos | WES | S, A, HPV | USA |
| Smith et al., 2019 | 0 | 21 | 0.00 | 1 | 21 | 4.76 | 0 | 21 | 0.00 | NP | Targeted NGS | S | USA |
| Westbrook et al., 2019 | | | | 1 | 23 | 4.35 | 0 | 23 | 0.00 | NP | Targeted NGS | S | USA |
| Samstein et al., 2019 | 5 | 139 | 3.60 | 3 | 139 | 2.16 | 3 | 139 | 2.16 | NP | Targeted NGS | NP | USA |
| Priestley et al., 2019 | 2 | 42 | 4.76 | 0 | 42 | 0.00 | 0 | 42 | 0.00 | NP | WGS | NP | Netherlands |
| Pérez Sayáns et al 2019 | 33 | 528 | 6.25 | 9 | 528 | 1.70 | 14 | 528 | 2.65 | Both | Targeted NGS, WGS, WES | S | Multi centered |
| Kobyashi et al., 2019 | 7 | 284 | 2.46 | | | | | | | Both | PCR+Direct seq | S, A | Japan |
| Dogan et al., 2019 | 6 | 25 | 24.00 | 0 | 25 | 0.00 | 0 | 25 | 0.00 | NP | Targeted NGS | S | USA |
| Gauthaman et al., 2020 | | | | 34 | 56 | 60.71 | | | | NP | PCR-RFLP | NP | India |
| Kyurkchiyan et al., 2020 | 3 | 57 | 5.26 | 2 | 57 | 3.51 | 1 | 57 | 1.75 | HPV Neg | Targeted NGS | NP | Bulgaria |

| | | | | | | | | | | | | | |
|---|---|---|---|---|---|---|---|---|---|---|---|---|---|
| Morita et al., 2020 | 2 | 101 | 1.98 | 7 | 101 | 6.93 | 0 | 101 | 0.00 | NP | SnapShot Multiplex assay | NP | Japan |
| Morfouace et al., 2020 | 0 | 14 | 0.00 | 0 | 14 | 0.00 | | | | NP | Targeted NGS | NP | France |
| Kawamura et al., 2020 | 0 | 20 | 0.00 | 0 | 20 | 0.00 | 0 | 20 | 0.00 | NP | PCR-SSCP | NP | Japan |
| Jayaprakash et al., 2020 | 6 | 28 | 21.43 | 0 | 28 | 0.00 | 0 | 28 | 0.00 | NP | Targeted NGS | NP | India |
| Leblanc et al., 2020 | 4 | 115 | 3.48 | 2 | 115 | 1.74 | 0 | 115 | 0.00 | Both | High Resolution Melting Analysis and sanger | NP | France |
| Mueller et al., 2020 | 14 | 63 | 22.22 | 3 | 63 | 4.76 | 0 | 63 | 0.00 | NP | Targeted NGS | S | Australia |
| Kim et al., 2020 | 4 | 42 | 9.52 | 0 | 42 | 0.00 | 0 | 42 | 0.00 | NP | Targeted NGS | S | Korea |
| Hsieh et al., 2020 | 9 | 33 | 27.27 | | | | 8 | 33 | 24.24 | NP | PCR+Direct seq | NP | Taiwan |
| ORCA ICGC | 21 | 178 | 11.80 | 6 | 178 | 3.37 | 2 | 178 | 1.12 | NP | targeted NGS, WGS | S, A, B | India |
| Masato et al., 2021 | 66 | 83 | 79.52 | 0 | 83 | 0.00 | 0 | 83 | 0.00 | NP | PCR+Direct seq | NP | Japan |
| Sanchez-Fernandez et al., 2021 | 0 | 48 | 0.00 | 7 | 48 | 14.58 | 2 | 48 | 4.17 | NP | Targeted NGS | S, A, W | Spain |
| AACR GENIE V9.1 | 55 | 1636 | 3.36 | 37 | 1636 | 2.26 | 20 | 1636 | 1.22 | NP | WES | S | Multi centered |
| Reder et al., 2021 | 9 | 56 | 16.07 | 8 | 56 | 14.29 | 7 | 56 | 12.50 | Both | Targeted NGS | S, A | Germany |
| Patel et al., 2021 | 4 | 30 | 13.33 | 0 | 30 | 0.00 | 0 | 30 | 0.00 | NP | WES | T | India |

NP-not provided, HPV pos- human papillomavirus positive, HPV neg- human papillomavirus negative, S-smoking T-Tabacco, A-Alcohol, B-betel chewing, W wood/leather dust, C- other chemical exposure, HPV-. human papillomavirus, EBV- Epstein–Barr virus

## Table S3: Detailed anatomical site data

| | HRAS | | | | | | | | | | | | | | | |
|---|---|---|---|---|---|---|---|---|---|---|---|---|---|---|---|---|
| | **Oral cavity** | | **Salivary gland** | | **Sinonasal** | | **Nasopharynx** | | **Oropharynx** | | **Hypopharynx** | | **Larynx** | | **Other** | |
| **cohort** | mutated | total | mutated | total | mutated | total | mutated | total | mutated | total | mutated | total | mutated | total | mutated | total |
| Das et al., 2000 | 14 | 50 | | | | | | | | | | | | | | |
| Yoo et al., 2000 | | | 5 | 24 | | | | | | | | | | | | |
| Yoo et al., 2000 | | | 9 | 50 | | | | | | | | | | | | |
| Weber et al., 2003 | | | | | | | | | | | | | | | | |
| Perrone et al., 2003 | | | | | 0 | 18 | | | | | | | | | | |
| Ruiz-Godoy et al., 2006 | | | | | | | | | | | | | | | | |
| Sathyan et al., 2006 | | | | | | | | | | | | | | | | |
| Frattini et al., 2006 | | | | | | | | | | | | | | | | |
| Augello et al., 2006 | | | 11 | 33 | | | | | | | | | | | | |
| Sathyan et al., 2007 | 19 | 152 | | | | | | | | | | | | | | |
| Gupta et al., 2007 | 0 | 20 | | | | | 0 | 1 | | | | | 0 | 14 | | |
| Sheikh Ali et al., 2008 | | | | | | | | | | | | | | | | |
| Chou et al., 2008 | | | | | | | | | | | | | | | | |
| Bornholdt et al., 2008 | | | | | | | | | | | | | | | | |
| Dahse et al., 2009 | | | | | | | | | | | | | | | | |
| Murugan et al., 2009 | 10 | 56 | | | | | | | | | | | | | | |
| Bruckman et al., 2010 | | | | | | | | | | | | | | | | |
| van Damme et al., 2010 | | | | | | | | | | | | | | | | |
| Chang et al., 2010 | 8 | 58 | | | | | | | | | | | | | | |
| Tetsu et al., 2010 | 7 | 8 | 0 | 3 | 0 | 1 | | | | | | | | | 3 | 5 |
| Murray et al., 2010 | | | | | | | | | | | | | | | | |
| Agarwal et al., 2011 | 4 | 75 | | | | | | | 1 | 21 | 0 | 9 | 0 | 13 | | |
| Stransky et al., 2011 | 3 | 51 | | | 0 | 2 | | | 0 | 15 | 0 | 9 | 1 | 15 | | |
| Cohen et al., 2011 | 0 | 37 | | | | | | | | | | | | | | |
| Trivedi et al., 2011 | | | | | | | | | | | | | | | | |
| Wang et al., 2011 | | | | | | | | | | | | | | | | |
| Friedland et al., 2011 | | | | | | | | | | | | | | | | |

| | | | | | | | | | | | | | | | | |
|---|---|---|---|---|---|---|---|---|---|---|---|---|---|---|---|---|
| Szabo et al., 2011 | | | | | | | | | | | | | | | | |
| Szanyi et al., 2011 | | | | | | | | | | | | | | | | |
| Suda et al., 2012 | | | | | | | | | | | | | | | | |
| Koumaki et al., 2012 | 3 | 86 | | | | | | | | | | | | | | |
| Lopez et al., 2012 and Garcia-Inclan et al., 2012 | | | | | | | | | | | | | | | | |
| Smilek et al., 2012 | | | | | | | | | | | | | | | | |
| Bissada et al., 2013 | | | | | | | | | | | | | | | | |
| Choisea et al., 2013 | | | | | | | | | 1 | 58 | | | | | | |
| Lechner et al., 2013 | | | | | | | | | 0 | 34 | | | | | | |
| Pickering et al., 2013 | 5 | 40 | | | | | | | | | | | | | | |
| Ho et al., 2013 | | | 5 | 59 | | | | | | | | | | | | |
| Stephens et al., 2013 | | | 0 | 24 | | | | | | | | | | | | |
| Liu et al., 2013 | | | | | | | | | | | | | | | | |
| Cros et al., 2013 | | | 7 | 107 | | | | | | | | | | | | |
| Fuji et al., 2013 | | | | | | | | | | | | | | | | |
| Projetti et al., 2013 | | | | | | | | | | | | | | | | |
| Szablewski et al., 2013 | | | | | | | | | | | | | | | | |
| Carvalho et al., 2013 | | | | | | | | | | | | | | | | |
| Zanaruddin et al., 2013 | 3 | 107 | | | | | | | | | | | | | | |
| Wetterskog et al., 2013 | | | | | | | | | | | | | | | | |
| Fury et al., 2013 | | | | | | | | | | | | | | | | |
| Chang et al., 2014 | 10 | 79 | | | | | | | | | | | | | | |
| Zhang et al., 2014 | | | | | | | | | | | | | | | | |
| Lin et al., 2014 | | | | | | | 0 | 56 | | | | | | | | |
| Ross et al., 2014 | | | 0 | 15 | | | | | | | | | | | | |
| Al Rawi et al., 2014 | | | | | | | | | | | | | | | | |
| Prigge et al., 2014 | | | | | | | | | | | | | | | | |
| Tan et al., 2014 | | | | | | | | | | | | | | | | |
| Chung et al., 2014 | | | | | | | | | | | | | | | | |
| Boeckx et al., 2014 | | | | | | | | | | | | | | | | |
| Choisea et al., 2014 | | | 4 | 14 | | | | | | | | | | | | |
| Lin et al., 2014 | | | | | | | | | | | | | | | | |

| | | | | | | | | | | | | | | | | |
|---|---|---|---|---|---|---|---|---|---|---|---|---|---|---|---|---|
| Zhang et al., 2014 | | | | | | | 1 | 123 | | | | | | | | |
| Franchi et al., 2014 | | | | | | | | | | | | | | | | |
| Zhang et al., 2015 | | | | | | | | | | | | | | | | |
| Seiwert et al., 2015 | | | | | | | | | | | | | | | | |
| Vettore et al., 2015 | 1 | 60 | | | | | | | | | | | | | | |
| Chen et al., 2015 | 32 | 345 | | | | | | | | | | | | | | |
| Fu et al., 2015 | | | | | | | | | | | | | | | | |
| Grunewald et al., 2015 | | | 20 | 84 | | | | | | | | | | | | |
| Kato et al., 2015 | | | 13 | 117 | | | | | | | | | | | | |
| Choisea et al., 2015 | | | 10 | 29 | | | | | | | | | | | | |
| Braig et al., 2016 | 3 | 12 | | | 0 | 2 | | | 1 | 19 | 1 | 9 | 0 | 4 | | |
| Rettig et al., 2016 | | | 0 | 25 | | | | | | | | | | | | |
| Mitani et al., 2016 | | | 0 | 65 | | | | | | | | | | | | |
| Drier et al., 2016 | | | 0 | 10 | | | | | | | | | | | | |
| Shalmon et al., 2016 | | | | | | | | | | | | | | | | |
| Wang et al., 2016 | | | 2 | 149 | | | | | | | | | | | | |
| Tinhofer et al., 2016 | | | | | | | | | | | | | | | | |
| Kucuk et al., 2016 | | | | | | | | | | | | | | | | |
| Schneider et al., 2016 | | | | | | | | | | | | | | | | |
| Choisea et al., 2016 | | | 13 | 38 | | | | | | | | | | | | |
| Dalin et al., 2016 | | | 7 | 31 | | | | | | | | | | | | |
| Udager et al., 2016 | | | | | | | | | | | | | | | | |
| Wu et al., 2016 | 4 | 56 | | | | | | | 0 | 76 | | | 4 | 82 | | |
| Chau et al., 2016 | 3 | 60 | | | 0 | 5 | 0 | 7 | 3 | 97 | 0 | 6 | 1 | 24 | 3 | 14 |
| Bell et al., 2016 | | | | | | | | | | | | | | | 0 | 24 |
| Luk et al., 2016 | | | 3 | 23 | | | | | | | | | | | | |
| Al-Hebshi et al., 2016 | | | | | | | | | | | | | | | | |
| Hedberg et al., 2016 | 0 | 6 | | | | | | | | | 0 | 3 | 0 | 4 | | |
| Chuerduangphui et al., 2017 | | | | | | | | | | | | | | | | |
| Morris et al., 2017 | 1 | 26 | 4 | 32 | 1 | 15 | 0 | 9 | 0 | 23 | 0 | 2 | 0 | 8 | 3 | 36 |
| Yue et al., 2017 | | | | | | | | | | | | | | | | |

| | | | | | | | | | | | | | | | | |
|---|---|---|---|---|---|---|---|---|---|---|---|---|---|---|---|---|
| Abdolkarim Moazeni-Roodi et al., 2017 | | | | | | | | | | | | | | | | |
| Zhang et al., 2017 | | | | | | | 1 | 94 | | | | | | | | |
| Ali et al., 2017 | | | | | | | | | | | | | | | | |
| Upadhyay et al., 2017 | 3 | 24 | | | | | | | | | | | | | | |
| kang et al., 2017 | | | 1 | 18 | | | | | | | | | | | | |
| Dalin et al., 2017 | | | 1 | 40 | | | | | | | | | | | | |
| Li et al., 2017 | | | | | | | 1 | 105 | | | | | | | | |
| Dogan et al., 2017 | | | | | 0 | 30 | | | | | | | | | | |
| Krishna et al., 2018 | | | | | | | | | | | | | | | | |
| Lin et al., 2018 | | | | | | | | | | | | | | | | |
| Saida et al., 2018 | | | 4 | 70 | | | | | | | | | | | | |
| Hallani et al., 2018 | | | 8 | 23 | | | | | | | | | | | | |
| Shimura et al., 2018 | | | 23 | 140 | | | | | | | | | | | | |
| Perdomo et al., 2018 | | | | | | | | | | | | | | | | |
| Vossen etal., 2018 | | | | | | | | | | | | | | | | |
| Batta et al., 2019 | 8 | 39 | | | | | | | | | | | | | 0 | 7 |
| Akagi et al., 2019 | | | | | | | | | | | | | | | | |
| Chung et al., 2019 | | | | | | | | | | | | | | | | |
| Reder et al., 2019 | | | | | | | | | 5 | 12 | | | | | | |
| Stanek et al., 2019 | | | | | | | | | | | | | | | | |
| Urano et al., 2019 | | | | | | | | | | | | | | | | |
| Nakaguro et al., 2019 | | | 7 | 21 | | | | | | | | | | | | |
| Wang et al., 2019 | | | | | | | | | | | | | | | | |
| Reder et al., 2019 | | | | | | | | | 0 | 12 | | | | | | |
| Gauthaman et al., 2020 | | | | | | | | | | | | | | | | |
| Kyurkchiyan et al., 2020 | | | | | | | | | | | | | | | | |
| ORCA ICGC | | | | | | | | | | | | | 3 | 57 | | |
| Sasaki et al., 2020 | 6 | 12 | | | | | | | | | 7 | 20 | 0 | 7 | 4 | 12 |
| Morita et al., 2020 | | | 2 | 101 | | | | | | | | | | | | |
| Morfouace et al., 2020 | | | | | 0 | 3 | 0 | 10 | | | | | | | 0 | 1 |
| Kawamura et al., 2020 | 0 | 20 | | | | | | | | | | | | | | |
| Pérez Sayáns et al 2019 | 28 | 303 | | | | | | | 1 | 82 | 0 | 10 | 1 | 117 | | |

| | | | | | | | | | | | | | | | | |
|---|---|---|---|---|---|---|---|---|---|---|---|---|---|---|---|---|
| Masato et al., 2021 | | | | | | | | | | | | | | | | |
| Sanchez-Fernandez et al., 2021 | | | | | 0 | 48 | | | | | | | | | | |
| AACR GENIE V9.0 | 21 | 468 | 38 | 791 | 3 | 80 | 0 | 82 | 4 | 306 | 0 | 29 | 4 | 98 | 0 | 21 |
| Dubot et al., 2018 | 0 | 61 | | | | | | | 1 | 22 | 0 | 17 | 1 | 22 | | |
| Zehir et al., 2017 | 4 | 59 | 1 | 9 | 1 | 12 | 0 | 17 | 0 | 44 | 0 | 3 | 0 | 16 | 0 | 26 |
| Zehir et al., 2017 SG | | | 5 | 105 | | | | | | | | | | | | |
| Bersani et al., 2017 | | | | | | | | | 5 | 325 | | | | | 0 | 19 |
| Biswas et al., 2014 | 7 | 84 | | | | | | | | | | | | | | |
| Haft et al., 2019 | | | | | | | | | 2 | 46 | | | | | | |
| Jayaprakash et al., 2019 | 6 | 28 | | | | | | | | | | | | | | |
| Mirghani et al., 2018 | | | | | | | | | 0 | 62 | | | | | | |
| Nakagaki et al., 2017 | 2 | 47 | | | | | | | | | | | | | | |
| Ock et al., 2016 | | | | | | | | | | | | | | | | |
| Oikawa et al., 2016 | 6 | 220 | | | | | | | | | | | | | | |
| Smith et al., 2019 | | | | | | | | | | | | | 0 | 21 | | |
| Su et al., 2017 | | | | | | | | | | | | | | | | |
| Westbrook et al., 2019 | | | | | | | | | | | | | | | | |
| Samstein et al., 2019 | 2 | 56 | | | 1 | 6 | 0 | 14 | 2 | 31 | 0 | 8 | 0 | 14 | 0 | 9 |
| Robinson et al., 2017 | 0 | 8 | | | 0 | 1 | | | 1 | 2 | | | 0 | 1 | 1 | 2 |
| Robinson et al., 2017 | | | 1 | 14 | | | | | | | | | | | | |
| Priestley et al., 2019 | 0 | 12 | 1 | 19 | 0 | 10 | 0 | 1 | 0 | 3 | 0 | 5 | 1 | 2 | 0 | 1 |
| Popovic et al., 2010 | 13 | 60 | | | | | | | | | | | | | 8 | 39 |
| Pickering et al., 2014 | | | | | | | | | | | | | | | | |
| Leblanc et al., 2020 | | | | | | | | | | | | | | | | |
| Mueller et al., 2020 | | | 14 | 63 | | | | | | | | | | | | |
| Kim et al., 2020 | | | 4 | 42 | | | | | | | | | | | | |
| Hsieh et al., 2020 | | | 9 | 33 | | | | | | | | | | | | |
| Khoo et al., 2017 | | | 4 | 15 | | | | | | | | | | | | |
| Nakagaki et al., 2018 | | | | | | | | | | | | | | | | |
| Reder et al., 2021 | | | | | | | | | 9 | 56 | | | | | | |
| Patel et al., 2021 | 4 | 30 | | | | | | | | | | | | | | |
| Fonseca et al., 2015 | | | 0 | 17 | | | | | | | | | | | | |
| Li et al., 2018 | | | | | | | 34 | 168 | | | | | | | | |

| | | | | | | | | | | | | | | | | |
|---|---|---|---|---|---|---|---|---|---|---|---|---|---|---|---|---|
| Lin et al., 2002 | | | | | | | | | | | | | 10 | 28 | | |
| Russo et al., 2006 | | | | | | | | | | | | | 5 | 81 | | |
| Kobyashi et al., 2019 | 6 | 153 | | | | | | | 1 | 32 | 0 | 74 | 0 | 25 | | |
| Dogan et al., 2019 | | | 6 | 25 | | | | | | | | | | | | |
| Ginkel et al., 2016 | 6 | 34 | | | | | | | 3 | 37 | 1 | 16 | 0 | 18 | 1 | 14 |
| Rampias et al., 2014 | | | | | | | | | 3 | 13 | 0 | 8 | 7 | 82 | 0 | 3 |

| | **KRAS** | | | | | | | | | | | | | | | |
|---|---|---|---|---|---|---|---|---|---|---|---|---|---|---|---|---|
| | **Oral cavity** | | **Salivary gland** | | **Sinonasal** | | **Nasopharynx** | | **Oropharynx** | | **Hypopharynx** | | **Larynx** | | **Other** | |
| Cohorts | mutated | total | mutated | total | mutated | total | mutated | total | mutated | total | mutated | total | mutated | total | mutated | total |
| Das et al., 2000 | 4 | 12 | | | | | | | | | | | | | | |
| Yoo et al., 2000 | | | 2 | 24 | | | | | | | | | | | | |
| Yoo et al., 2000 | | | | | | | | | | | | | | | | |
| Weber et al., 2003 | 1 | 13 | | | | | | | 1 | 33 | 2 | 18 | 1 | 25 | | |
| Perrone et al., 2003 | | | | | | | | | | | | | | | | |
| Ruiz-Godoy et al., 2006 | | | | | | | | | | | | | 0 | 20 | | |
| Sathyan et al., 2006 | | | | | | | | | | | | | | | | |
| Frattini et al., 2006 | | | | | 9 | 18 | | | | | | | | | | |
| Augello et al., 2006 | | | 2 | 33 | | | | | | | | | | | | |
| Sathyan et al., 2007 | 0 | 152 | | | | | | | | | | | | | | |
| Gupta et al., 2007 | 0 | 20 | | | | | 0 | 1 | | | | | 0 | 14 | | |
| Sheikh Ali et al., 2008 | 0 | 51 | | | | | | | 0 | 9 | 0 | 14 | 0 | 17 | | |
| Chou et al., 2008 | | | | | | | 0 | 45 | | | | | | | | |
| Bornholdt et al., 2008 | | | | | 8 | 174 | | | | | | | | | | |
| Dahse et al., 2009 | | | 1 | 65 | | | | | | | | | | | | |
| Murugan et al., 2009 | | | | | | | | | | | | | | | | |
| Bruckman et al., 2010 | 1 | 42 | | | | | | | | | | | | | | |
| van Damme et al., 2010 | | | | | | | | | 1 | 22 | | | | | | |
| Chang et al., 2010 | 0 | 58 | | | | | | | | | | | | | | |
| Tetsu et al., 2010 | 1 | 8 | 0 | 3 | 0 | 1 | | | | | | | | | 0 | 5 |
| Murray et al., 2010 | | | | | | | | | | | | | | | | |

| | | | | | | | | | | | | | | | | |
|---|---|---|---|---|---|---|---|---|---|---|---|---|---|---|---|---|
| Agarwal et al., 2011 | 0 | 75 | | | | | | | 0 | 21 | 0 | 9 | 0 | 13 | | |
| Stransky et al., 2011 | 0 | 51 | | | 0 | 2 | | | 0 | 15 | 1 | 9 | 0 | 15 | | |
| Cohen et al., 2011 | 0 | 37 | | | | | | | | | | | | | | |
| Trivedi et al., 2011 | | | | | | | | | | | | | | | | |
| Wang et al., 2011 | 0 | 47 | | | | | | | | | | | | | | |
| Friedland et al., 2011 | | | | | | | | | 0 | 20 | | | | | | |
| Szabo et al., 2011 | | | | | | | | | | | | | | | | |
| Szanyi et al., 2011 | | | | | | | | | | | | | | | | |
| Suda et al., 2012 | 2 | 31 | | | 0 | 11 | | | 1 | 25 | 0 | 25 | 0 | 23 | | |
| Koumaki et al., 2012 | | | | | | | | | | | | | | | | |
| Lopez et al., 2012 and Garcia-Inclan et al., 2012 | | | | | 7 | 58 | | | | | | | | | | |
| Smilek et al., 2012 | | | | | | | | | | | | | | | | |
| Bissada et al., 2013 | 0 | 21 | | | | | | | 4 | 123 | 0 | 11 | 3 | 29 | 0 | 11 |
| Choisea et al., 2013 | | | | | | | | | | | | | | | | |
| Lechner et al., 2013 | | | | | ` | | | | 2 | 34 | | | | | | |
| Pickering et al., 2013 | 0 | 40 | | | | | | | | | | | | | | |
| Ho et al., 2013 | | | 0 | 59 | | | | | | | | | | | | |
| Stephens et al., 2013 | | | 0 | 24 | | | | | | | | | | | | |
| Liu et al., 2013 | | | | | | | | | | | | | | | | |
| Cros et al., 2013 | | | 4 | 107 | | | | | | | | | | | | |
| Fuji et al., 2013 | | | | | | | | | 0 | 71 | 0 | 83 | 0 | 51 | | |
| Projetti et al., 2013 | | | | | 2 | 34 | | | | | | | | | | |
| Szablewski et al., 2013 | | | | | 12 | 28 | | | | | | | | | | |
| Carvalho et al., 2013 | 0 | 66 | | | | | | | 0 | 12 | 0 | 2 | 0 | 14 | | |
| Zanaruddin et al., 2013 | 0 | 107 | | | | | | | | | | | | | | |
| Wetterskog et al., 2013 | | | | | | | | | | | | | | | | |
| Fury et al., 2013 | 1 | 1 | | | 0 | 1 | 0 | 1 | 0 | 14 | 0 | 1 | | | | |
| Chang et al., 2014 | 0 | 79 | | | | | | | | | | | | | | |
| Zhang et al., 2014 | | | | | | | | | | | | | | | | |
| Lin et al., 2014 | | | | | | | 1 | 56 | | | | | | | | |
| Ross et al., 2014 | | | 0 | 15 | | | | | | | | | | | | |
| Al Rawi et al., 2014 | | | | | | | | | | | | | | | | |

| | | | | | | | | | | | | | | | | |
|---|---|---|---|---|---|---|---|---|---|---|---|---|---|---|---|---|
| Prigge et al., 2014 | 0 | 3 | | | | | | | 0 | 21 | 0 | 1 | | | | |
| Tan et al., 2014 | 0 | 66 | | | | | | | | | | | | | | |
| Chung et al., 2014 | | | | | | | | | | | | | | | | |
| Boeckx et al., 2014 | | | | | | | | | | | | | | | | |
| Choisea et al., 2014 | | | | | | | | | | | | | | | | |
| Lin et al., 2014 | | | | | | | 1 | 66 | | | | | | | | |
| Zhang et al., 2014 | | | | | | | 0 | 123 | | | | | | | | |
| Franchi et al., 2014 | | | | | 1 | 27 | | | | | | | | | | |
| Zhang et al., 2015 | | | | | | | 1 | 70 | | | | | | | | |
| Seiwert et al., 2015 | | | | | | | | | | | | | | | | |
| Vettore et al., 2015 | | | | | | | | | | | | | | | | |
| Chen et al., 2015 | 8 | 345 | | | | | | | | | | | | | | |
| Fu et al., 2015 | | | 2 | 18 | | | | | | | | | | | | |
| Grunewald et al., 2015 | | | 0 | 84 | | | | | | | | | | | | |
| Kato et al., 2015 | | | | | | | | | | | | | | | | |
| Choisea et al., 2015 | | | 0 | 29 | | | | | | | | | | | | |
| Braig et al., 2016 | 0 | 12 | | | 0 | 2 | | | 0 | 19 | 1 | 9 | 1 | 4 | | |
| Rettig et al., 2016 | | | 0 | 25 | | | | | | | | | | | | |
| Mitani et al., 2016 | | | 0 | 65 | | | | | | | | | | | | |
| Drier et al., 2016 | | | 0 | 10 | | | | | | | | | | | | |
| Shalmon et al., 2016 | | | 0 | 21 | | | | | | | | | | | | |
| Wang et al., 2016 | | | 0 | 149 | | | | | | | | | | | | |
| Tinhofer et al., 2016 | | | | | | | | | | | | | | | | |
| Kucuk et al., 2016 | 0 | 26 | | | | | | | | | | | | | | |
| Schneider et al., 2016 | | | 0 | 43 | | | | | | | | | | | | |
| Choisea et al., 2016 | | | | | | | | | | | | | | | | |
| Dalin et al., 2016 | | | 0 | 31 | | | | | | | | | | | | |
| Udager et al., 2016 | | | | | 56 | 56 | | | | | | | | | | |
| Wu et al., 2016 | 1 | 56 | | | | | | | 8 | 76 | | | 9 | 82 | | |
| Chau et al., 2016 | 1 | 60 | | | 0 | 5 | 0 | 7 | 0 | 97 | 0 | 6 | 0 | 24 | 0 | 14 |
| Bell et al., 2016 | | | | | | | | | | | | | | | 10 | 24 |
| Luk et al., 2016 | | | 0 | 23 | | | | | | | | | | | | |

| | | | | | | | | | | | | | | | | |
|---|---|---|---|---|---|---|---|---|---|---|---|---|---|---|---|---|
| Al-Hebshi et al., 2016 | | | | | | | | | | | | | | | | |
| Hedberg et al., 2016 | 0 | 6 | | | | | | | | | 0 | 3 | 1 | 4 | | |
| Chuerduangphui et al., 2017 | | | | | | | | | | | | | | | | |
| Morris et al., 2017 | 1 | 26 | 4 | 32 | 1 | 15 | 0 | 9 | 0 | 23 | 0 | 2 | 0 | 8 | 3 | 36 |
| Yue et al., 2017 | | | | | | | | | | | | | | | | |
| Abdolkarim Moazeni-Roodi et al., 2017 | | | | | | | | | | | | | | | | |
| Zhang et al., 2017 | | | | | | | 3 | 94 | | | | | | | | |
| Ali et al., 2017 | | | | | | | | | | | | | | | | |
| Upadhyay et al., 2017 | 0 | 25 | | | | | | | | | | | | | | |
| kang et al., 2017 | | | 0 | 18 | | | | | | | | | | | | |
| Dalin et al., 2017 | | | 0 | 40 | | | | | | | | | | | | |
| Li et al., 2017 | | | | | | | 1 | 105 | | | | | | | | |
| Dogan et al., 2017 | | | | | 3 | 30 | | | | | | | | | | |
| Krishna et al., 2018 | | | | | | | | | | | | | | | | |
| Lin et al., 2018 | | | | | | | | | | | | | | | | |
| Saida et al., 2018 | | | 6 | 70 | | | | | | | | | | | | |
| Hallani et al., 2018 | | | 0 | 23 | | | | | | | | | | | | |
| Shimura et al., 2018 | | | 0 | 140 | | | | | | | | | | | | |
| Perdomo et al., 2018 | | | | | | | | | | | | | | | | |
| Vossen etal., 2018 | | | | | | | | | | | | | | | | |
| Batta et al., 2019 | 0 | 39 | | | | | | | | | | | | | 0 | 7 |
| Akagi et al., 2019 | | | | | | | | | | | | | | | | |
| Chung et al., 2019 | | | | | | | | | | | | | | | | |
| Reder et al., 2019 | | | | | | | | | 0 | 12 | | | | | | |
| Stanek et al., 2019 | | | | | | | | | | | | | | | | |
| Urano et al., 2019 | | | | | | | | | | | | | | | | |
| Nakaguro et al., 2019 | | | | | | | | | | | | | | | | |
| Wang et al., 2019 | | | | | 30 | 80 | | | | | | | | | | |
| Reder et al., 2019 | | | | | | | | | 1 | 12 | | | | | | |
| Gauthaman et al., 2020 | 25 | 44 | | | | | | | 2 | 3 | 4 | 6 | 0 | 0 | 3 | 4 |
| Kyurkchiyan et al., 2020 | | | | | | | | | | | | | 2 | 57 | | |

| | | | | | | | | | | | | | | | | |
|---|---|---|---|---|---|---|---|---|---|---|---|---|---|---|---|---|
| ORCA ICGC | | | | | | | | | | | | | | | | |
| Sasaki et al., 2020 | 1 | 12 | | | | | | | | | 11 | 20 | 2 | 7 | 4 | 12 |
| Morita et al., 2020 | | | 7 | 101 | | | | | | | | | | | | |
| Morfouace et al., 2020 | | | | | 0 | 3 | 0 | 10 | | | | | | | 0 | 1 |
| Kawamura et al., 2020 | 0 | 20 | | | | | | | | | | | | | | |
| Pérez Sayáns et al 2019 | 1 | 303 | | | | | | | 0 | 82 | 0 | 10 | 0 | 117 | | |
| Masato et al., 2021 | | | | | | | | | | | | | | | | |
| Sanchez-Fernandez et al., 2021 | | | | | | | | | | | | | | | 7 | 48 |
| AACR GENIE V9.0 | 8 | 468 | 8 | 791 | 0 | 80 | 2 | 82 | 4 | 306 | 0 | 29 | 4 | 98 | 0 | 21 |
| Dubot et al., 2018 | 0 | 61 | | | | | | | 2 | 22 | 0 | 17 | 0 | 22 | | |
| Zehir et al., 2017 | 1 | 59 | 0 | 9 | 0 | 12 | 0 | 17 | 0 | 52 | 0 | 3 | 1 | 16 | 0 | 18 |
| Zehir et al., 2017 SG | | | 1 | 105 | | | | | | | | | | | | |
| Bersani et al., 2017 | | | | | | | | | 9 | 325 | | | | | 0 | 19 |
| Biswas et al., 2014 | | | | | | | | | | | | | | | | |
| Haft et al., 2019 | | | | | | | | | 0 | 46 | | | | | | |
| Jayaprakash et al., 2019 | 0 | 28 | | | | | | | | | | | | | | |
| Mirghani et al., 2018 | | | | | | | | | 2 | 62 | | | | | | |
| Nakagaki et al., 2017 | 0 | 47 | | | | | | | | | | | | | | |
| Ock et al., 2016 | | | | | | | | | | | | | | | | |
| Oikawa et al., 2016 | 1 | 220 | | | | | | | | | | | | | | |
| Smith et al., 2019 | | | | | | | | | | | | | | | | |
| Su et al., 2017 | | | | | | | | | | | | | 1 | 21 | | |
| Westbrook et al., 2019 | 0 | 6 | | | | | 0 | 1 | 0 | 7 | 0 | 2 | 1 | 7 | | |
| Samstein et al., 2019 | 1 | 56 | | | 0 | 6 | 0 | 14 | 1 | 33 | 0 | 8 | 1 | 14 | 0 | 9 |
| Robinson et al., 2017 | 0 | 8 | | | 0 | 1 | | | 0 | 2 | | | 0 | 1 | 1 | 2 |
| Robinson et al., 2017 | | | 0 | 14 | | | | | | | | | | | | |
| Priestley et al., 2019 | 0 | 12 | 0 | 19 | 0 | 10 | 0 | 1 | 0 | 3 | 0 | 5 | 0 | 2 | 0 | 1 |
| Popovic et al., 2010 | | | | | | | | | | | | | | | 0 | 39 |
| Pickering et al., 2014 | | | | | | | | | | | | | | | | |
| Leblanc et al., 2020 | | | | | | | | | | | | | | | | |
| Mueller et al., 2020 | | | 3 | 63 | | | | | | | | | | | | |
| Kim et al., 2020 | | | 0 | 42 | | | | | | | | | | | | |
| Hsieh et al., 2020 | | | | | | | | | | | | | | | | |

| | | | | | | | | | | | | | | | | |
|---|---|---|---|---|---|---|---|---|---|---|---|---|---|---|---|---|
| Khoo et al., 2017 | | | 4 | 15 | | | | | | | | | | | | |
| Nakagaki et al., 2018 | | | | | | | | | | | | | | | | |
| Reder et al., 2021 | | | | | | | | | 8 | 56 | | | | | | |
| Patel et al., 2021 | 0 | 30 | | | | | | | | | | | | | | |
| Fonseca et al., 2015 | | | 3 | 17 | | | | | | | | | | | | |
| Li et al., 2018 | | | | | | | | | | | | | | | | |
| Lin et al., 2002 | | | | | | | | | | | | | | | | |
| Russo et al., 2006 | | | | | | | | | | | | | 0 | 81 | | |
| Kobyashi et al., 2019 | | | | | | | | | | | | | | | | |
| Dogan et al., 2019 | | | 0 | 25 | | | | | | | | | | | | |
| Ginkel et al., 2016 | | | | | | | | | 1 | 37 | | | | | 1 | 14 |
| Rampias et al., 2014 | | | | | | | | | | | | | | | | |

| | **NRAS** | | | | | | | | | | | | | | | |
|---|---|---|---|---|---|---|---|---|---|---|---|---|---|---|---|---|
| | **Oral cavity** | | **Salivary gland** | | **Sinonasal** | | **Nasopharynx** | | **Oropharynx** | | **Hypopharynx** | | **Larynx** | | **Other** | |
| Cohorts | mutated | total | mutated | total | mutated | total | mutated | total | mutated | total | mutated | total | mutated | total | mutated | total |
| Das et al., 2000 | | | | | | | | | | | | | | | | |
| Yoo et al., 2000 | | | | | | | | | | | | | | | | |
| Yoo et al., 2000 | | | | | | | | | | | | | | | | |
| Weber et al., 2003 | | | | | | | | | | | | | | | | |
| Perrone et al., 2003 | | | | | | | | | | | | | | | | |
| Ruiz-Godoy et al., 2006 | | | | | | | | | | | | | | | | |
| Sathyan et al., 2006 | | | | | | | | | | | | | | | | |
| Frattini et al., 2006 | | | | | | | | | | | | | | | | |
| Augello et al., 2006 | | | | | | | | | | | | | | | | |
| Sathyan et al., 2007 | 0 | 152 | | | | | | | | | | | | | | |
| Gupta et al., 2007 | 0 | 20 | | | | | 0 | 1 | | | | | 0 | 14 | | |
| Sheikh Ali et al., 2008 | | | | | | | | | | | | | | | | |
| Chou et al., 2008 | | | | | | | | | | | | | | | | |
| Bornholdt et al., 2008 | | | | | | | | | | | | | | | | |
| Dahse et al., 2009 | | | | | | | | | | | | | | | | |

| | | | | | | | | | | | | | | | | |
|---|---|---|---|---|---|---|---|---|---|---|---|---|---|---|---|---|
| Murugan et al., 2009 | | | | | | | | | | | | | | | | |
| Bruckman et al., 2010 | | | | | | | | | | | | | | | | |
| van Damme et al., 2010 | | | | | | | | | | | | | | | | |
| Chang et al., 2010 | 0 | 58 | | | | | | | | | | | | | | |
| Tetsu et al., 2010 | 0 | 8 | 1 | 3 | 0 | 1 | | | | | | | | | 0 | 5 |
| Murray et al., 2010 | | | | | | | | | | | | | | | | |
| Agarwal et al., 2011 | 0 | 75 | | | | | | | 0 | 21 | 0 | 9 | 0 | 13 | | |
| Stransky et al., 2011 | 0 | 51 | | | 0 | 2 | | | 0 | 15 | 0 | 9 | 0 | 15 | | |
| Cohen et al., 2011 | 0 | 37 | | | | | | | | | | | | | | |
| Trivedi et al., 2011 | | | | | | | | | | | | | | | | |
| Wang et al., 2011 | | | | | | | | | | | | | | | | |
| Friedland et al., 2011 | | | | | | | | | | | | | | | | |
| Szabo et al., 2011 | | | | | | | | | | | | | | | | |
| Szanyi et al., 2011 | | | | | | | | | | | | | | | | |
| Suda et al., 2012 | | | | | | | | | | | | | | | | |
| Koumaki et al., 2012 | | | | | | | | | | | | | | | | |
| Lopez et al., 2012 and Garcia-Inclan et al., 2012 | | | | | | | | | | | | | | | | |
| Smilek et al., 2012 | | | | | | | | | | | | | | | | |
| Bissada et al., 2013 | | | | | | | | | | | | | | | | |
| Choisea et al., 2013 | | | | | | | | | | | | | | | | |
| Lechner et al., 2013 | | | | | | | | | 0 | 34 | | | | | | |
| Pickering et al., 2013 | 0 | 40 | | | | | | | | | | | | | | |
| Ho et al., 2013 | | | 1 | 59 | | | | | | | | | | | | |
| Stephens et al., 2013 | | | 0 | 24 | | | | | | | | | | | | |
| Liu et al., 2013 | | | | | | | | | | | | | | | | |
| Cros et al., 2013 | | | 2 | 107 | | | | | | | | | | | | |
| Fuji et al., 2013 | | | | | | | | | | | | | | | | |
| Projetti et al., 2013 | | | | | | | | | | | | | | | | |
| Szablewski et al., 2013 | | | | | | | | | | | | | | | | |
| Carvalho et al., 2013 | | | | | | | | | | | | | | | | |
| Zanaruddin et al., 2013 | 0 | 107 | | | | | | | | | | | | | | |

| | | | | | | | | | | | | | | | | |
|---|---|---|---|---|---|---|---|---|---|---|---|---|---|---|---|---|
| Wetterskog et al., 2013 | | | | | | | | | | | | | | | | |
| Fury et al., 2013 | 1 | 1 | | | 0 | 1 | 0 | 1 | 0 | 14 | 0 | 1 | | | | |
| Chang et al., 2014 | 0 | 79 | | | | | | | | | | | | | | |
| Zhang et al., 2014 | | | | | | | | | | | | | | | | |
| Lin et al., 2014 | | | | | | | 1 | 56 | | | | | | | | |
| Ross et al., 2014 | | | 0 | 15 | | | | | | | | | | | | |
| Al Rawi et al., 2014 | | | | | | | | | | | | | | | | |
| Prigge et al., 2014 | | | | | | | | | | | | | | | | |
| Tan et al., 2014 | | | | | | | | | | | | | | | | |
| Chung et al., 2014 | | | | | | | | | | | | | | | | |
| Boeckx et al., 2014 | | | | | | | | | | | | | | | | |
| Choisea et al., 2014 | | | | | | | | | | | | | | | | |
| Lin et al., 2014 | | | | | | | 0 | 66 | | | | | | | | |
| Zhang et al., 2014 | | | | | | | 5 | 123 | | | | | | | | |
| Franchi et al., 2014 | | | | | | | | | | | | | | | | |
| Zhang et al., 2015 | | | | | | | | | | | | | | | | |
| Seiwert et al., 2015 | | | | | | | | | | | | | | | | |
| Vettore et al., 2015 | | | | | | | | | | | | | | | | |
| Chen et al., 2015 | | | | | | | | | | | | | | | | |
| Fu et al., 2015 | | | 0 | 18 | | | | | | | | | | | | |
| Grunewald et al., 2015 | | | 2 | 84 | | | | | | | | | | | | |
| Kato et al., 2015 | | | | | | | | | | | | | | | | |
| Choisea et al., 2015 | | | 0 | 29 | | | | | | | | | | | | |
| Braig et al., 2016 | 1 | 12 | | | 0 | 2 | | | 0 | 19 | 0 | 9 | 1 | 4 | | |
| Rettig et al., 2016 | | | 1 | 25 | | | | | | | | | | | | |
| Mitani et al., 2016 | | | 0 | 65 | | | | | | | | | | | | |
| Drier et al., 2016 | | | 0 | 10 | | | | | | | | | | | | |
| Shalmon et al., 2016 | | | | | | | | | | | | | | | | |
| Wang et al., 2016 | | | 0 | 149 | | | | | | | | | | | | |
| Tinhofer et al., 2016 | | | | | | | | | | | | | | | | |
| Kucuk et al., 2016 | | | | | | | | | | | | | | | | |
| Schneider et al., 2016 | | | | | | | | | | | | | | | | |

| | | | | | | | | | | | | | | | | |
|---|---|---|---|---|---|---|---|---|---|---|---|---|---|---|---|---|
| Choisea et al., 2016 | | | | | | | | | | | | | | | | |
| Dalin et al., 2016 | | | 0 | 31 | | | | | | | | | | | | |
| Udager et al., 2016 | | | | | | | | | | | | | | | | |
| Wu et al., 2016 | | | | | | | | | | | | | | | | |
| Chau et al., 2016 | 2 | 60 | | | 0 | 5 | 0 | 7 | 0 | 97 | 0 | 6 | 0 | 24 | 0 | 14 |
| Bell et al., 2016 | | | | | | | | | | | | | | | 2 | 24 |
| Luk et al., 2016 | | | 1 | 23 | | | | | | | | | | | | |
| Al-Hebshi et al., 2016 | | | | | | | | | | | | | | | | |
| Hedberg et al., 2016 | 1 | 6 | | | | | | | | | 0 | 3 | 0 | 4 | | |
| Chuerduangphui et al., 2017 | | | | | | | | | | | | | | | | |
| Morris et al., 2017 | 1 | 26 | 0 | 32 | 0 | 15 | 1 | 9 | 0 | 23 | 0 | 2 | 0 | 8 | 0 | 36 |
| Yue et al., 2017 | | | | | | | | | | | | | | | | |
| Abdolkarim Moazeni-Roodi et al., 2017 | | | | | | | | | | | | | | | | |
| Zhang et al., 2017 | | | | | | | 1 | 94 | | | | | | | | |
| Ali et al., 2017 | | | | | | | | | | | | | | | | |
| Upadhyay et al., 2017 | 0 | 24 | | | | | | | | | | | | | | |
| kang et al., 2017 | | | 0 | 18 | | | | | | | | | | | | |
| Dalin et al., 2017 | | | 0 | 40 | | | | | | | | | | | | |
| Li et al., 2017 | | | | | | | 3 | 105 | | | | | | | | |
| Dogan et al., 2017 | | | | | 0 | 30 | | | | | | | | | | |
| Krishna et al., 2018 | | | | | | | | | | | | | | | | |
| Lin et al., 2018 | | | | | | | | | | | | | | | | |
| Saida et al., 2018 | | | 0 | 70 | | | | | | | | | | | | |
| Perdomo et al., 2018 | | | | | | | | | | | | | | | | |
| Vossen etal., 2018 | | | | | | | | | | | | | | | | |
| Hallani et al., 2018 | | | 0 | 23 | | | | | | | | | | | | |
| Shimura et al., 2018 | | | 0 | 140 | | | | | | | | | | | | |
| Batta et al., 2019 | 0 | 39 | | | | | | | | | | | | | 0 | 7 |
| Akagi et al., 2019 | | | | | | | | | | | | | | | | |
| Chung et al., 2019 | | | | | | | | | | | | | | | | |
| Reder et al., 2019 | | | | | | | | | | | | | | | | |

| | | | | | | | | | | | | | | | | |
|---|---|---|---|---|---|---|---|---|---|---|---|---|---|---|---|---|
| Stanek et al., 2019 | | | | | | | | | 1 | 12 | | | | | | |
| Urano et al., 2019 | | | | | | | | | | | | | | | | |
| Nakaguro et al., 2019 | | | | | | | | | | | | | | | | |
| Wang et al., 2019 | | | | | | | | | | | | | | | | |
| Reder et al., 2019 | | | | | | | | | 0 | 12 | | | | | | |
| Gauthaman et al., 2020 | | | | | | | | | | | | | | | | |
| Kyurkchiyan et al., 2020 | | | | | | | | | | | | | 1 | 57 | | |
| ORCA ICGC | | | | | | | | | | | | | | | | |
| Sasaki et al., 2020 | | | | | | | | | | | | | | | | |
| Morita et al., 2020 | | | 0 | 101 | | | | | | | | | | | | |
| Morfouace et al., 2020 | | | | | 0 | 3 | 0 | 10 | | | | | | | 0 | 1 |
| Kawamura et al., 2020 | 0 | 20 | | | | | | | | | | | | | | |
| Pérez Sayáns et al 2019 | 0 | 303 | | | | | | | 0 | 82 | 0 | 10 | 1 | 117 | | |
| Masato et al., 2021 | | | | | | | | | | | | | | | | |
| Sanchez-Fernandez et al., 2021 | | | | | | | | | | | | | | | 2 | 48 |
| AACR GENIE V9.0 | 5 | 468 | 5 | 791 | 1 | 80 | 1 | 82 | 3 | 306 | 0 | 29 | 0 | 98 | 0 | 21 |
| Dubot et al., 2018 | 1 | 61 | | | | | | | 0 | 22 | 0 | 17 | 0 | 22 | | |
| Zehir et al., 2017 | 1 | 59 | 0 | 9 | 0 | 12 | 1 | 17 | 0 | 52 | 0 | 3 | 0 | 16 | 1 | 18 |
| Zehir et al., 2017 SG | | | 1 | 105 | | | | | | | | | | | | |
| Bersani et al., 2017 | | | | | | | | | 4 | 325 | | | | | 0 | 19 |
| Biswas et al., 2014 | | | | | | | | | | | | | | | | |
| Haft et al., 2019 | | | | | | | | | 0 | 46 | | | | | | |
| Jayaprakash et al., 2019 | 0 | 28 | | | | | | | | | | | | | | |
| Mirghani et al., 2018 | | | | | | | | | 0 | 62 | | | | | | |
| Nakagaki et al., 2017 | 0 | 47 | | | | | | | | | | | | | | |
| Ock et al., 2016 | | | | | | | | | | | | | | | | |
| Oikawa et al., 2016 | 2 | 220 | | | | | | | | | | | | | | |
| Smith et al., 2019 | | | | | | | | | | | | | 0 | 21 | | |
| Su et al., 2017 | | | | | | | | | | | | | | | | |
| Westbrook et al., 2019 | 0 | 6 | | | | | 0 | 1 | 0 | 7 | 0 | 2 | 0 | 7 | | |
| Samstein et al., 2019 | 1 | 56 | | | 0 | 6 | 1 | 14 | 1 | 33 | 0 | 8 | 0 | 14 | 0 | 9 |

| | | | | | | | | | | | | | | | | |
|---|---|---|---|---|---|---|---|---|---|---|---|---|---|---|---|---|
| Robinson et al., 2017 | 0 | 8 | | | 0 | 1 | | | 0 | 2 | | | 0 | 1 | 0 | 2 |
| Robinson et al., 2017 | | | 0 | 14 | | | | | | | | | | | | |
| Priestley et al., 2019 | 0 | 12 | 0 | 19 | 0 | 10 | 0 | 1 | 0 | 3 | 0 | 5 | 0 | 2 | 0 | 1 |
| Popovic et al., 2010 | | | | | | | | | | | | | | | 2 | 39 |
| Pickering et al., 2014 | | | | | | | | | | | | | | | | |
| Leblanc et al., 2020 | | | | | | | | | | | | | | | | |
| Mueller et al., 2020 | | | 0 | 63 | | | | | | | | | | | | |
| Kim et al., 2020 | | | 0 | 42 | | | | | | | | | | | | |
| Hsieh et al., 2020 | | | 8 | 33 | | | | | | | | | | | | |
| Khoo et al., 2017 | | | 4 | 15 | | | | | | | | | | | | |
| Nakagaki et al., 2018 | | | | | | | | | | | | | | | | |
| Reder et al., 2021 | | | | | | | | | 7 | 56 | | | | | | |
| Patel et al., 2021 | 0 | 30 | | | | | | | | | | | | | | |
| Fonseca et al., 2015 | | | 1 | 17 | | | | | | | | | | | | |
| Li et al., 2018 | | | | | | | | | | | | | | | | |
| Lin et al., 2002 | | | | | | | | | | | | | | | | |
| Russo et al., 2006 | | | | | | | | | | | | | 0 | 81 | | |
| Kobyashi et al., 2019 | | | | | | | | | | | | | | | | |
| Dogan et al., 2019 | | | 0 | 25 | | | | | | | | | | | | |
| Ginkel et al., 2016 | | | | | | | | | | | | | | | | |
| Rampias et al., 2014 | | | | | | | | | | | | | | | | |
