# Supplementary material for: Worldwide Prevalence and Clinical Characteristics of RAS Mutations in Head and Neck Cancer: A Systematic Review and Meta-Analysis
Source: Front Oncol. 2022 May 6;12:838911. doi: 10.3389/fonc.2022.838911 (PMC9121358; doi:10.3389/fonc.2022.838911)

Figure S2: Forest plot of RAS mutation frequency according to geographical region

Forest plot of RAS mutation frequency [%] in head and neck cancer according to geographical region. CI: Confidence interval. I^2: Inconsistency index.

Prevalence of HRAS Mutation

Study	Cases	Total	Prevalence (%)	95% C.I.
**Geographical Region: North America**				
Drier et al., 2016	0	10	0.00	[0.28; 44.83]
Hedberg et al., 2016	0	12	0.00	[0.24; 40.32]
Robinson et al., 2017	1	14	7.14	[1.00; 37.03]
Ross et al., 2014	0	15	0.00	[0.19; 35.03]
Robinson et al., 2017	2	15	13.33	[3.36; 40.54]
Fonseca et al., 2015	0	17	0.00	[0.17; 32.21]
Tetsu et al., 2010	8	17	47.06	[25.54; 69.73]
Kang et al., 2017	1	18	5.56	[0.78; 30.65]
Smith et al., 2019	0	21	0.00	[0.14; 27.74]
Hallani et al., 2018	8	23	34.78	[18.44; 55.71]
Bell et al., 2016	0	24	0.00	[0.12; 25.13]
Rettig et al., 2016	0	25	0.00	[0.12; 24.36]
Dogan et al., 2019	6	25	24.00	[11.20; 44.16]
Choisea et al., 2015	10	29	34.48	[19.66; 53.09]
Dogan et al., 2017	0	30	0.00	[0.10; 21.14]
Dalin et al., 2016	7	31	22.58	[11.16; 40.37]
Pickering et al., 2014	8	39	20.51	[10.60; 35.96]
Dalin et al., 2017	1	40	2.50	[0.35; 15.73]
Pickering et al., 2013	5	40	12.50	[5.30; 26.72]
Haft et al., 2019	2	46	4.35	[1.09; 15.79]
Wetterskog et al., 2013	1	48	2.08	[0.29; 13.36]
Zhang et al., 2014	1	53	1.89	[0.27; 12.21]
Ho et al., 2013	5	59	8.47	[3.57; 18.80]
Mitani et al., 2016	0	65	0.00	[0.05; 10.98]
Stransky et al., 2011	4	74	5.41	[2.04; 13.53]
Li et al., 2017	1	105	0.95	[0.13; 6.45]
Zehir et al., 2017	5	105	4.76	[2.00; 10.93]
Seiwert et al., 2015	4	120	3.33	[1.26; 8.54]
Agarwal et al., 2011	5	120	4.17	[1.74; 9.62]
Samstein et al., 2019	5	139	3.60	[1.51; 8.35]
Wang et al., 2016	21	149	14.09	[9.37; 20.65]
Liu et al., 2013	7	151	4.64	[2.23; 9.40]
Morris et al., 2017	9	151	5.96	[3.13; 11.06]
Zehir et al., 2017	6	178	3.37	[1.52; 7.30]
Chau et al., 2016	10	213	4.69	[2.54; 8.50]
Feldman et al., 2016	8	299	2.68	[1.34; 5.26]
**Fixed effect model**		**2520**	**8.58**	**[7.34; 10.01]**
**Random effects model**			**6.87**	**[4.77; 9.79]**
Heterogeneity: $I^2 = 76\%$, $\tau^2 = 0.8730$, $\chi^2_{35} = 145.23$ ($p < 0.01$)				
**Geographical Region: Europe**				
Stephens et al., 2013	0	24	0.00	[0.12; 25.13]
Reder et al., 2019	5	24	20.83	[8.95; 41.34]
Lechner et al., 2013	0	34	0.00	[0.09; 19.12]
Priestley et al., 2019	2	42	4.76	[1.19; 17.14]
Braig et al., 2016	5	46	10.87	[4.60; 23.58]
Sanchez-Fernandez et al., 2021	0	48	0.00	[0.06; 14.32]
Reder et al., 2021	9	56	16.07	[8.58; 28.09]
Kyurkchiyan et al., 2020	3	57	5.26	[1.71; 15.09]
Mirghani et al., 2018	0	62	0.00	[0.05; 11.45]
Russo et al., 2006	5	81	6.17	[2.59; 13.99]
Grunewald et al., 2015	20	84	23.81	[15.91; 34.05]
Cros et al., 2013	7	107	6.54	[3.15; 13.09]
Vossen etal., 2018	2	111	1.80	[0.45; 6.92]
Leblanc et al., 2020	4	115	3.48	[1.31; 8.90]
Dubot et al.,2018	2	122	1.64	[0.41; 6.32]
Tinhofer et al., 2016	1	179	0.56	[0.08; 3.86]
Bersani et al., 2017	5	344	1.45	[0.61; 3.44]
**Fixed effect model**		**1536**	**8.15**	**[6.48; 10.20]**
**Random effects model**			**4.65**	**[2.57; 8.28]**
Heterogeneity: $I^2 = 80\%$, $\tau^2 = 1.1592$, $\chi^2_{16} = 80.58$ ($p < 0.01$)				
**Geographical Region: South Asia**				
Upadhyay et al., 2017	3	25	12.00	[3.92; 31.30]
Jayaprakash et al., 2020	6	28	21.43	[9.96; 40.21]
Patel et al., 2021	4	30	13.33	[5.10; 30.59]
Batta et al., 2019	8	46	17.39	[8.94; 31.09]
Das et al., 2000	14	50	28.00	[17.34; 41.89]
Sathyan et al., 2007	19	152	12.50	[8.12; 18.77]
ORCA ICGC	21	178	11.80	[7.82; 17.42]
**Fixed effect model**		**509**	**15.28**	**[12.34; 18.77]**
**Random effects model**			**15.84**	**[11.84; 20.88]**
Heterogeneity: $I^2 = 39\%$, $\tau^2 = 0.0757$, $\chi^2_{6} = 9.77$ ($p = 0.13$)				
**Geographical Region: East Asia**				
Kawamura et al., 2020	0	20	0.00	[0.15; 28.74]
Chung et al., 2019	0	33	0.00	[0.09; 19.59]
Kim et al., 2020	4	42	9.52	[3.62; 22.78]
Nakagaki et al., 2017	2	47	4.26	[1.07; 15.48]
Lin et al., 2014	0	56	0.00	[0.05; 12.53]
Chang et al., 2010	8	58	13.79	[7.05; 25.23]
Saida et al., 2018	5	70	7.14	[3.00; 16.04]
Ock et al., 2016	26	71	36.62	[26.28; 48.36]
Chang et al., 2014	10	79	12.66	[6.95; 21.95]
Nakagaki et al., 2018	4	80	5.00	[1.89; 12.58]
Masato et al., 2021	66	83	79.52	[69.49; 86.87]
Zhang et al., 2017	1	94	1.06	[0.15; 7.16]
Morita et al., 2020	2	101	1.98	[0.50; 7.57]
Zanaruddin et al., 2013	3	107	2.80	[0.91; 8.33]
Zhang et al., 2014	1	123	0.81	[0.11; 5.54]
Ali et al., 2017	1	190	0.53	[0.07; 3.64]
Oikawa et al.,2016	4	220	1.82	[0.68; 4.74]
**Fixed effect model**		**1474**	**17.74**	**[14.63; 21.34]**
**Random effects model**			**5.07**	**[1.99; 12.31]**
Heterogeneity: $I^2 = 93\%$, $\tau^2 = 3.5220$, $\chi^2_{16} = 240.6$ ($p < 0.01$)				
**Fixed effect model**		**6039**	**11.20**	**[10.17; 12.31]**
**Random effects model**			**6.56**	**[4.90; 8.72]**
Heterogeneity: $I^2 = 86\%$, $\tau^2 = 1.3881$, $\chi^2_{76} = 524.60$ ($p < 0.01$)				
Residual heterogeneity: $I^2 = 85\%$, $\chi^2_{73} = 476.18$ ($p < 0.01$)				

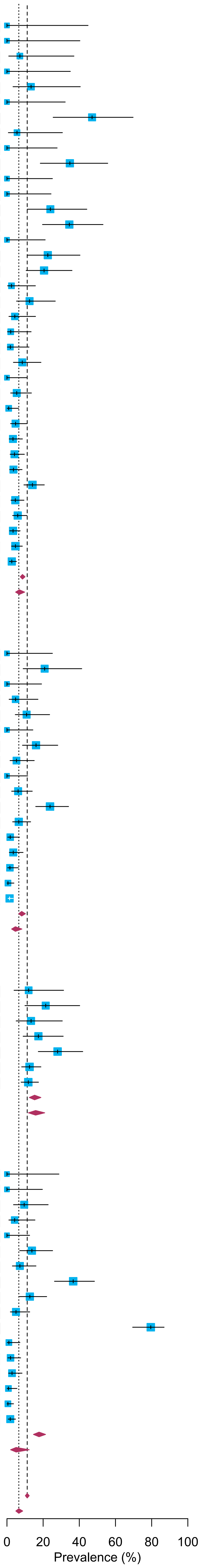

Prevalence of KRAS Mutation

Study	Cases	Total	Prevalence (%)	95% C.I.
**Geographical Region: North America**				
Hedberg et al., 2016	1	12	8.33	[1.16; 41.32]
Robinson et al., 2017	0	14	0.00	[0.21; 36.63]
Ross et al., 2014	0	15	0.00	[0.19; 35.03]
Robinson et al., 2017	1	15	6.67	[0.93; 35.20]
Tetsu et al., 2010	1	17	5.88	[0.82; 32.03]
Fonseca et al., 2015	3	17	17.65	[5.80; 42.71]
Kang et al., 2017	0	18	0.00	[0.16; 30.96]
Drier et al., 2016	0	19	0.00	[0.15; 29.81]
Smith et al., 2019	1	21	4.76	[0.67; 27.14]
Hallani et al., 2018	0	23	0.00	[0.13; 25.94]
Bell et al., 2016	10	24	41.67	[24.09; 61.66]
Rettig et al., 2016	0	25	0.00	[0.12; 24.36]
Dogan et al., 2019	0	25	0.00	[0.12; 24.36]
Choisea et al., 2015	0	29	0.00	[0.10; 21.72]
Dalin et al., 2016	0	31	0.00	[0.10; 20.60]
Pickering et al., 2014	0	39	0.00	[0.08; 17.08]
Pickering et al., 2013	0	40	0.00	[0.08; 16.72]
Dalin et al., 2017	0	40	0.00	[0.08; 16.72]
Dogan et al., 2017	3	40	7.50	[2.44; 20.82]
Haft et al., 2019	0	46	0.00	[0.07; 14.86]
Wetterskog et al., 2013	0	48	0.00	[0.06; 14.32]
Zhang et al., 2014	6	53	11.32	[5.18; 22.99]
Ho et al., 2013	0	59	0.00	[0.05; 11.97]
Mitani et al., 2016	0	65	0.00	[0.05; 10.98]
Stransky et al., 2011	1	74	1.35	[0.19; 8.97]
Li et al., 2017	1	105	0.95	[0.13; 6.45]
Zehir et al., 2017	1	105	0.95	[0.13; 6.45]
Agarwal et al., 2011	0	120	0.00	[0.03; 6.25]
Seiwert et al., 2015	4	120	3.33	[1.26; 8.54]
Samstein et al., 2019	3	139	2.16	[0.70; 6.48]
Wang et al., 2016	0	149	0.00	[0.02; 5.10]
Liu et al., 2013	2	151	1.32	[0.33; 5.14]
Morris et al., 2017	2	151	1.32	[0.33; 5.14]
Zehir et al., 2017	2	178	1.12	[0.28; 4.38]
Chau et al., 2016	1	213	0.47	[0.07; 3.25]
Feldman et al., 2016	11	448	2.46	[1.36; 4.38]
Fixed effect model		2688	3.94	[3.06; 5.06]
Random effects model			2.61	[1.60; 4.23]
Heterogeneity: I^2 = 65%, τ^2 = 1.2403, χ^2_{35} = 99.73 (p < 0.01)				
**Geographical Region: Europe**				
Stephens et al., 2013	0	24	0.00	[0.12; 25.13]
Reder et al., 2019	1	24	4.17	[0.58; 24.35]
Lechner et al., 2013	2	34	5.88	[1.48; 20.68]
Priestley et al., 2019	0	42	0.00	[0.07; 16.05]
Braig et al., 2016	2	46	4.35	[1.09; 15.79]
Sanchez-Fernandez et al., 2021	7	48	14.58	[7.11; 27.57]
Reder et al., 2021	8	56	14.29	[7.31; 26.05]
Kyurkchiyan et al., 2020	2	57	3.51	[0.88; 12.97]
Mirghani et al., 2018	2	62	3.23	[0.81; 12.00]
Russo et al., 2006	0	81	0.00	[0.04; 9.00]
Grunewald et al., 2015	0	84	0.00	[0.04; 8.71]
Cros et al., 2013	4	107	3.74	[1.41; 9.54]
Vossen etal., 2018	0	111	0.00	[0.03; 6.73]
Leblanc et al., 2020	2	115	1.74	[0.44; 6.68]
Dubot et al.,2018	2	122	1.64	[0.41; 6.32]
Tinhofer et al., 2016	5	179	2.79	[1.17; 6.53]
Bersani et al., 2017	9	344	2.62	[1.37; 4.95]
Fixed effect model		1536	4.43	[3.35; 5.84]
Random effects model			3.54	[2.18; 5.71]
Heterogeneity: I^2 = 58%, τ^2 = 0.5373, χ^2_{16} = 37.72 (p < 0.01)				
**Geographical Region: South Asia**				
Das et al., 2000	4	12	33.33	[13.09; 62.41]
Upadhyay et al., 2017	0	25	0.00	[0.12; 24.36]
Jayaprakash et al., 2020	0	28	0.00	[0.11; 22.32]
Patel et al., 2021	0	30	0.00	[0.10; 21.14]
Batta et al., 2019	0	46	0.00	[0.07; 14.86]
Sathyan et al., 2007	0	152	0.00	[0.02; 5.00]
ORCA ICGC	6	178	3.37	[1.52; 7.30]
Fixed effect model		471	4.94	[2.79; 8.59]
Random effects model			2.95	[0.76; 10.77]
Heterogeneity: I^2 = 72%, τ^2 = 2.1599, χ^2_{6} = 21.32 (p < 0.01)				
**Geographical Region: East Asia**				
Kawamura et al., 2020	0	20	0.00	[0.15; 28.74]
Chung et al., 2019	1	33	3.03	[0.43; 18.61]
Kim et al., 2020	0	42	0.00	[0.07; 16.05]
Nakagaki et al., 2017	0	47	0.00	[0.06; 14.59]
Lin et al., 2014	1	56	1.79	[0.25; 11.61]
Chang et al., 2010	0	58	0.00	[0.05; 12.15]
Saida et al., 2018	6	70	8.57	[3.90; 17.79]
Ock et al., 2016	13	71	18.31	[10.94; 29.03]
Chang et al., 2014	0	79	0.00	[0.04; 9.21]
Nakagaki et al., 2018	0	80	0.00	[0.04; 9.10]
Masato et al., 2021	0	83	0.00	[0.04; 8.80]
Zhang et al., 2017	3	94	3.19	[1.03; 9.43]
Morita et al., 2020	7	101	6.93	[3.34; 13.83]
Zanaruddin et al., 2013	0	107	0.00	[0.03; 6.96]
Zhang et al., 2014	0	123	0.00	[0.03; 6.11]
Ali et al., 2017	3	190	1.58	[0.51; 4.78]
Oikawa et al.,2016	1	220	0.45	[0.06; 3.15]
Fixed effect model		1474	5.48	[4.01; 7.44]
Random effects model			2.20	[1.09; 4.42]
Heterogeneity: I^2 = 70%, τ^2 = 1.2652, χ^2_{16} = 54.08 (p < 0.01)				
**Fixed effect model**		**6169**	**4.50**	**[3.86; 5.24]**
**Random effects model**			**2.82**	**[2.09; 3.80]**
Heterogeneity: I^2 = 65%, τ^2 = 0.9708, χ^2_{76} = 215.60 (p < 0.01)				
Residual heterogeneity: I^2 = 66%, χ^2_{73} = 212.86 (p < 0.01)				

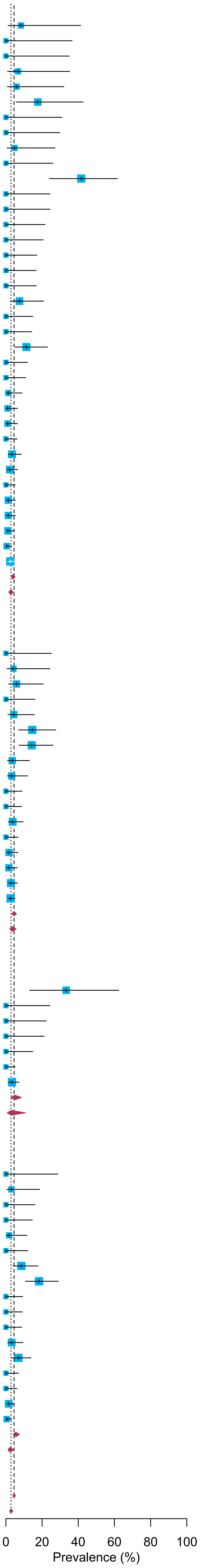

Prevalence of NRAS Mutation

Study	Cases	Total	Prevalence (%)	95% C.I.
**Geographical Region: North America**				
Drier et al., 2016	0	10	0.00	[0.28; 44.83]
Hedberg et al., 2016	1	12	8.33	[1.16; 41.32]
Robinson et al., 2017 salivary	0	14	0.00	[0.21; 36.63]
Ross et al., 2014	0	15	0.00	[0.19; 35.03]
Robinson et al., 2017	0	15	0.00	[0.19; 35.03]
Tetsu et al., 2010	1	17	5.88	[0.82; 32.03]
Fonseca et al., 2015	1	17	5.88	[0.82; 32.03]
kang et al., 2017	0	18	0.00	[0.16; 30.96]
Smith et al., 2019	0	21	0.00	[0.14; 27.74]
Hallani et al., 2018	0	23	0.00	[0.13; 25.94]
Bell et al., 2016	2	24	8.33	[2.09; 27.88]
Dogan et al., 2019	0	25	0.00	[0.12; 24.36]
Rettig et al., 2016	1	25	4.00	[0.56; 23.55]
Choisea et al., 2015	0	29	0.00	[0.10; 21.72]
Dogan et al., 2017	0	30	0.00	[0.10; 21.14]
Dalin et al., 2016	0	31	0.00	[0.10; 20.60]
Pickering et al., 2014	2	39	5.13	[1.29; 18.32]
Pickering et al., 2013	0	40	0.00	[0.08; 16.72]
Dalin et al., 2017	0	40	0.00	[0.08; 16.72]
Haft et al., 2019	0	46	0.00	[0.07; 14.86]
Wetterskog et al., 2013	0	48	0.00	[0.06; 14.32]
Zhang et al., 2014	6	53	11.32	[5.18; 22.99]
Ho et al., 2013	1	59	1.69	[0.24; 11.07]
Mitani et al., 2016	0	65	0.00	[0.05; 10.98]
Stransky et al., 2011	0	74	0.00	[0.04; 9.77]
Zehir et al., 2017	1	105	0.95	[0.13; 6.45]
Li et al., 2017	3	105	2.86	[0.92; 8.48]
Agarwal et al., 2011	0	120	0.00	[0.03; 6.25]
Seiwert et al., 2015	1	120	0.83	[0.12; 5.67]
Samstein et al., 2019	3	139	2.16	[0.70; 6.48]
Wang et al., 2016	0	149	0.00	[0.02; 5.10]
Liu et al., 2013	0	151	0.00	[0.02; 5.03]
Morris et al., 2017	2	151	1.32	[0.33; 5.14]
Zehir et al., 2017	3	178	1.69	[0.54; 5.09]
Chau et al., 2016	2	213	0.94	[0.23; 3.67]
Feldman et al., 2016	4	381	1.05	[0.39; 2.76]
**Fixed effect model**		**2602**	**2.45**	**[1.82; 3.28]**
**Random effects model**			**2.34**	**[1.69; 3.22]**
Heterogeneity: $I^2 = 10\%$, $\tau^2 = 0.1014$, $\chi^2_{35} = 39.06$ ($p = 0.29$)				
**Geographical Region: Europe**				
Stephens et al., 2013	0	24	0.00	[0.12; 25.13]
Reder et al., 2019	1	24	4.17	[0.58; 24.35]
Lechner et al., 2013	0	34	0.00	[0.09; 19.12]
Priestley et al., 2019	0	42	0.00	[0.07; 16.05]
Braig et al., 2016	2	46	4.35	[1.09; 15.79]
Sanchez-Fernandez et al., 2021	2	48	4.17	[1.04; 15.19]
Reder et al., 2021	7	56	12.50	[6.08; 23.98]
Kyurkchiyan et al., 2020	1	57	1.75	[0.25; 11.43]
Mirghani et al., 2018	0	62	0.00	[0.05; 11.45]
Russo et al., 2006	0	81	0.00	[0.04; 9.00]
Grunewald et al., 2015	2	84	2.38	[0.60; 9.02]
Cros et al., 2013	2	107	1.87	[0.47; 7.16]
Vossen etal., 2018	1	111	0.90	[0.13; 6.11]
Leblanc et al., 2020	0	115	0.00	[0.03; 6.51]
Dubot et al.,2018	1	122	0.82	[0.12; 5.58]
Tinhofer et al., 2016	2	179	1.12	[0.28; 4.36]
Bersani et al., 2017	4	344	1.16	[0.44; 3.06]
**Fixed effect model**		**1536**	**2.72**	**[1.88; 3.92]**
**Random effects model**			**2.08**	**[1.20; 3.60]**
Heterogeneity: $I^2 = 46\%$, $\tau^2 = 0.5642$, $\chi^2_{16} = 29.42$ ($p = 0.02$)				
**Geographical Region: South Asia**				
Upadhyay et al., 2017	0	25	0.00	[0.12; 24.36]
Jayaprakash et al., 2020	0	28	0.00	[0.11; 22.32]
Patel et al., 2021	0	30	0.00	[0.10; 21.14]
Batta et al., 2019	0	46	0.00	[0.07; 14.86]
Das et al., 2000	0	50	0.00	[0.06; 13.83]
Sathyan et al., 2007	0	152	0.00	[0.02; 5.00]
ORCA ICGC	2	178	1.12	[0.28; 4.38]
**Fixed effect model**		**509**	**1.11**	**[0.46; 2.64]**
**Random effects model**			**1.11**	**[0.46; 2.64]**
Heterogeneity: $I^2 = 0\%$, $\tau^2 = 0$, $\chi^2_{6} = 1.08$ ($p = 0.98$)				
**Geographical Region: East Asia**				
Kawamura et al., 2020	0	20	0.00	[0.15; 28.74]
Chung et al., 2019	0	33	0.00	[0.09; 19.59]
Kim et al., 2020	0	42	0.00	[0.07; 16.05]
Nakagaki et al., 2017	0	47	0.00	[0.06; 14.59]
Lin et al., 2014	1	56	1.79	[0.25; 11.61]
Chang et al., 2010	0	58	0.00	[0.05; 12.15]
Saida et al., 2018	0	70	0.00	[0.04; 10.27]
Chang et al., 2014	0	79	0.00	[0.04; 9.21]
Nakagaki et al., 2018	0	80	0.00	[0.04; 9.10]
Masato et al., 2021	0	83	0.00	[0.04; 8.80]
Zhang et al., 2017	1	94	1.06	[0.15; 7.16]
Morita et al., 2020	0	101	0.00	[0.03; 7.35]
Zanaruddin et al., 2013	0	107	0.00	[0.03; 6.96]
Zhang et al., 2014	5	123	4.07	[1.70; 9.39]
Ali et al., 2017	4	190	2.11	[0.79; 5.47]
Oikawa et al.,2016	2	220	0.91	[0.23; 3.56]
**Fixed effect model**		**1403**	**1.66**	**[1.05; 2.60]**
**Random effects model**			**1.66**	**[1.05; 2.60]**
Heterogeneity: $I^2 = 0\%$, $\tau^2 = 0$, $\chi^2_{15} = 9.18$ ($p = 0.87$)				
**Fixed effect model**		**6050**	**2.24**	**[1.83; 2.74]**
**Random effects model**			**2.08**	**[1.66; 2.59]**
Heterogeneity: $I^2 = 11\%$, $\tau^2 = 0.1046$, $\chi^2_{75} = 84.37$ ($p = 0.21$)				
Residual heterogeneity: $I^2 = 9\%$, $\chi^2_{72} = 78.75$ ($p = 0.27$)				

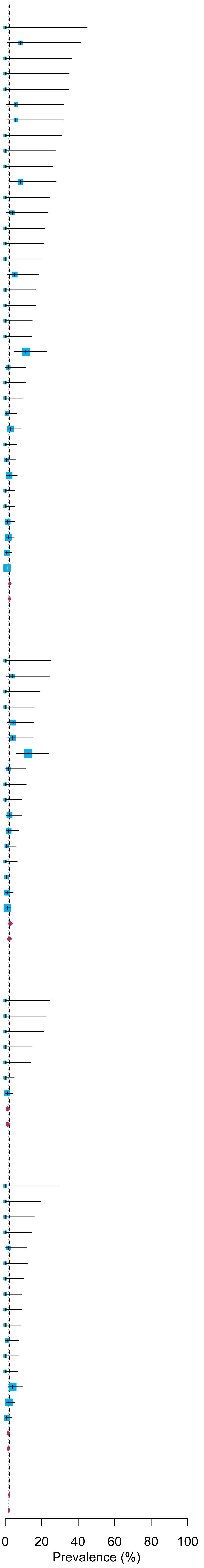

Figure S3: Mutation prevalence according to anatomical site

Forest plot of RAS mutation frequency [%] in head and neck cancer according to tumor anatomical site of origin. CI: Confidence interval. I^2: Inconsistency index.

HRAS Mutation in Different Anatomic Sites

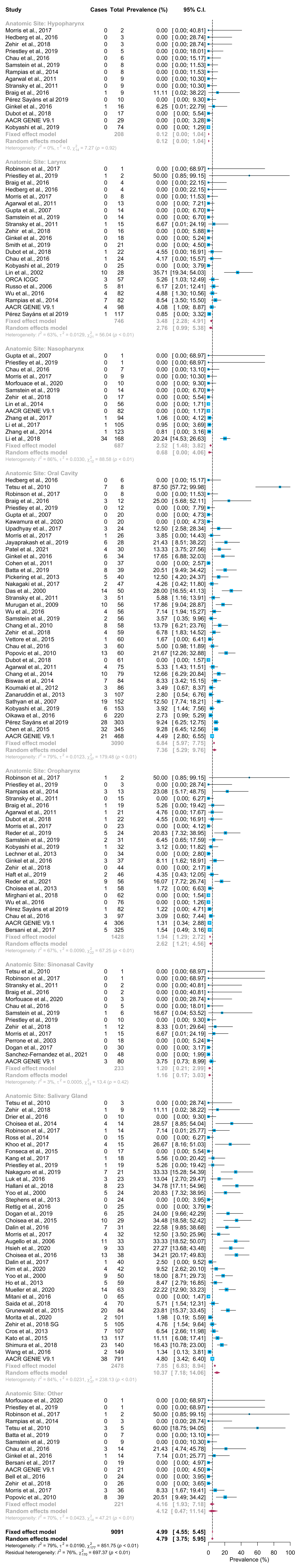

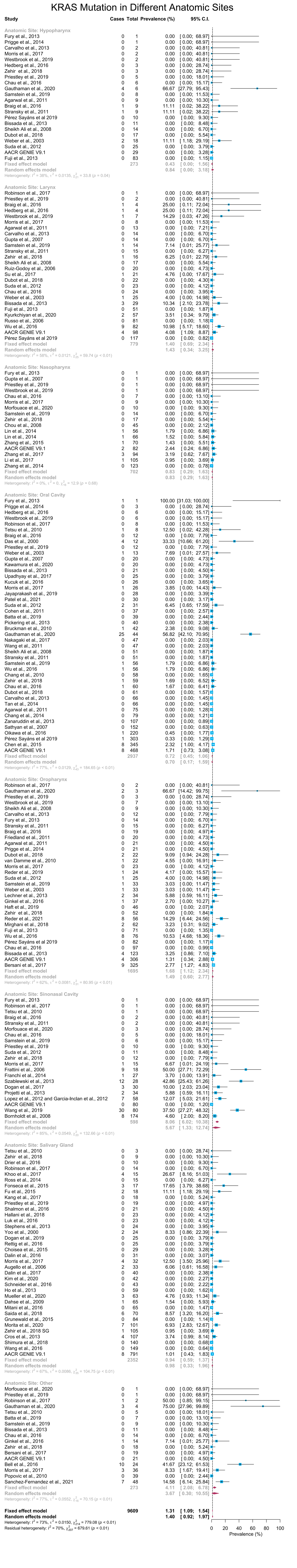
KRAS Mutation in Different Anatomic Sites
Study Cases Total Prevalence (%) 95% C.I.
Anatomic Site: Hypopharynx
Fury et al., 2013 0 1 0.00 [0.00; 68.97]
Prigge et al., 2014 0 1 0.00 [0.00; 68.97]
Carvalho et al., 2013 0 2 0.00 [0.00; 40.81]
Morris et al., 2017 0 2 0.00 [0.00; 40.81]
Westbrook et al., 2019 0 2 0.00 [0.00; 40.81]
Hedberg et al., 2016 0 3 0.00 [0.00; 28.74]
Zehir et al., 2018 0 3 0.00 [0.00; 28.74]
Priestley et al., 2019 0 5 0.00 [0.00; 18.01]
Chau et al., 2016 0 6 0.00 [0.00; 15.17]
Gauthaman et al., 2020 4 6 66.67 [27.79; 95.43]
Samstein et al., 2019 0 8 0.00 [0.00; 11.53]
Agarwal et al., 2011 0 9 0.00 [0.00; 10.30]
Braig et al., 2016 1 9 11.11 [0.02; 38.22]
Stransky et al., 2011 1 9 11.11 [0.02; 38.22]
Pérez Sayáns et al 2019 0 10 0.00 [0.00; 9.30]
Bissada et al., 2013 0 11 0.00 [0.00; 8.48]
Sheikh Ali et al., 2008 0 14 0.00 [0.00; 6.70]
Dubot et al., 2018 0 17 0.00 [0.00; 5.54]
Weber et al., 2003 2 18 11.11 [1.18; 29.19]
Suda et al., 2012 0 25 0.00 [0.00; 3.79]
AACR GENIE V9.1 0 29 0.00 [0.00; 3.28]
Fuji et al., 2013 0 83 0.00 [0.00; 1.15]
Fixed effect model 273 0.43 [0.00; 1.56]
Random effects model 0.84 [0.00; 3.18]
Heterogeneity: $I^2 = 38\%$, $\tau^2 = 0.0135$, $\chi^2_{21} = 33.8$ ($p = 0.04$)
Anatomic Site: Larynx
Robinson et al., 2017 0 1 0.00 [0.00; 68.97]
Priestley et al., 2019 0 2 0.00 [0.00; 40.81]
Braig et al., 2016 1 4 25.00 [0.11; 72.04]
Hedberg et al., 2016 1 4 25.00 [0.11; 72.04]
Westbrook et al., 2019 1 7 14.29 [0.03; 47.26]
Morris et al., 2017 0 8 0.00 [0.00; 11.53]
Agarwal et al., 2011 0 13 0.00 [0.00; 7.21]
Carvalho et al., 2013 0 14 0.00 [0.00; 6.70]
Gupta et al., 2007 0 14 0.00 [0.00; 6.70]
Samstein et al., 2019 1 14 7.14 [0.01; 25.77]
Stransky et al., 2011 0 15 0.00 [0.00; 6.27]
Zehir et al., 2018 1 16 6.25 [0.01; 22.79]
Sheikh Ali et al., 2008 0 17 0.00 [0.00; 5.54]
Ruiz-Godoy et al., 2006 0 20 0.00 [0.00; 4.73]
Su et al., 2017 1 21 4.76 [0.00; 17.67]
Dubot et al., 2018 0 22 0.00 [0.00; 4.30]
Suda et al., 2012 0 23 0.00 [0.00; 4.12]
Chau et al., 2016 0 24 0.00 [0.00; 3.95]
Weber et al., 2003 1 25 4.00 [0.00; 14.98]
Bissada et al., 2013 3 29 10.34 [2.10; 23.78]
Fuji et al., 2013 0 51 0.00 [0.00; 1.87]
Kyurkchiyan et al., 2020 2 57 3.51 [0.34; 9.79]
Russo et al., 2006 0 81 0.00 [0.00; 1.18]
Wu et al., 2016 9 82 10.98 [5.17; 18.60]
AACR GENIE V9.1 4 98 4.08 [1.09; 8.87]
Pérez Sayáns et al 2019 0 117 0.00 [0.00; 0.82]
Fixed effect model 779 1.40 [0.69; 2.34]
Random effects model 1.43 [0.34; 3.25]
Heterogeneity: $I^2 = 58\%$, $\tau^2 = 0.0121$, $\chi^2_{25} = 59.74$ ($p < 0.01$)
Anatomic Site: Nasopharynx
Fury et al., 2013 0 1 0.00 [0.00; 68.97]
Gupta et al., 2007 0 1 0.00 [0.00; 68.97]
Priestley et al., 2019 0 1 0.00 [0.00; 68.97]
Westbrook et al., 2019 0 1 0.00 [0.00; 68.97]
Chau et al., 2016 0 7 0.00 [0.00; 13.10]
Morris et al., 2017 0 9 0.00 [0.00; 10.30]
Morfouace et al., 2020 0 10 0.00 [0.00; 9.30]
Samstein et al., 2019 0 14 0.00 [0.00; 6.70]
Zehir et al., 2018 0 17 0.00 [0.00; 5.54]
Chou et al., 2008 0 45 0.00 [0.00; 2.12]
Lin et al., 2014 1 56 1.79 [0.00; 6.86]
Lin et al., 2014 1 66 1.52 [0.00; 5.84]
Zhang et al., 2015 1 70 1.43 [0.00; 5.51]
AACR GENIE V9.1 2 82 2.44 [0.24; 6.86]
Zhang et al., 2017 3 94 3.19 [0.62; 7.67]
Li et al., 2017 1 105 0.95 [0.00; 3.69]
Zhang et al., 2014 0 123 0.00 [0.00; 0.78]
Fixed effect model 702 0.83 [0.29; 1.63]
Random effects model 0.83 [0.29; 1.63]
Heterogeneity: $I^2 = 0\%$, $\tau^2 = 0$, $\chi^2_{16} = 12.9$ ($p = 0.68$)
Anatomic Site: Oral Cavity
Fury et al., 2013 1 1 100.00 [31.03; 100.00]
Prigge et al., 2014 0 3 0.00 [0.00; 28.74]
Hedberg et al., 2016 0 6 0.00 [0.00; 15.17]
Westbrook et al., 2019 0 6 0.00 [0.00; 15.17]
Robinson et al., 2017 0 8 0.00 [0.00; 11.53]
Tetsu et al., 2010 1 8 12.50 [0.02; 42.28]
Braig et al., 2016 0 12 0.00 [0.00; 7.79]
Das et al., 2000 4 12 33.33 [10.66; 61.20]
Priestley et al., 2019 0 12 0.00 [0.00; 7.79]
Weber et al., 2003 1 13 7.69 [0.01; 27.57]
Gupta et al., 2007 0 20 0.00 [0.00; 4.73]
Kawamura et al., 2020 0 20 0.00 [0.00; 4.73]
Bissada et al., 2013 0 21 0.00 [0.00; 4.50]
Upadhyay et al., 2017 0 25 0.00 [0.00; 3.79]
Kucuk et al., 2016 0 26 0.00 [0.00; 3.65]
Morris et al., 2017 1 26 3.85 [0.00; 14.43]
Jayaprakash et al., 2019 0 28 0.00 [0.00; 3.39]
Patel et al., 2021 0 30 0.00 [0.00; 3.17]
Suda et al., 2012 2 31 6.45 [0.65; 17.59]
Cohen et al., 2011 0 37 0.00 [0.00; 2.57]
Batta et al., 2019 0 39 0.00 [0.00; 2.44]
Pickering et al., 2013 0 40 0.00 [0.00; 2.38]
Bruckman et al., 2010 1 42 2.38 [0.00; 9.08]
Gauthaman et al., 2020 25 44 56.82 [42.10; 70.95]
Nakagaki et al., 2017 0 47 0.00 [0.00; 2.03]
Wang et al., 2011 0 47 0.00 [0.00; 2.03]
Sheikh Ali et al., 2008 0 51 0.00 [0.00; 1.87]
Stransky et al., 2011 0 51 0.00 [0.00; 1.87]
Samstein et al., 2019 1 56 1.79 [0.00; 6.86]
Wu et al., 2016 1 56 1.79 [0.00; 6.86]
Chang et al., 2010 0 58 0.00 [0.00; 1.65]
Zehir et al., 2018 1 59 1.69 [0.00; 6.52]
Chau et al., 2016 1 60 1.67 [0.00; 6.41]
Dubot et al., 2018 0 61 0.00 [0.00; 1.57]
Carvalho et al., 2013 0 66 0.00 [0.00; 1.45]
Tan et al., 2014 0 66 0.00 [0.00; 1.45]
Agarwal et al., 2011 0 75 0.00 [0.00; 1.28]
Chang et al., 2014 0 79 0.00 [0.00; 1.21]
Zanaruddin et al., 2013 0 107 0.00 [0.00; 0.89]
Sathyan et al., 2007 0 152 0.00 [0.00; 0.63]
Oikawa et al., 2016 1 220 0.45 [0.00; 1.77]
Pérez Sayáns et al 2019 1 303 0.33 [0.00; 1.29]
Chen et al., 2015 8 345 2.32 [1.00; 4.17]
AACR GENIE V9.1 8 468 1.71 [0.73; 3.08]
Fixed effect model 2937 0.72 [0.45; 1.06]
Random effects model 0.70 [0.17; 1.59]
Heterogeneity: $I^2 = 77\%$, $\tau^2 = 0.0129$, $\chi^2_{43} = 184.65$ ($p < 0.01$)
Anatomic Site: Oropharynx
Robinson et al., 2017 0 2 0.00 [0.00; 40.81]
Gauthaman et al., 2020 2 3 66.67 [14.42; 99.75]
Priestley et al., 2019 0 3 0.00 [0.00; 28.74]
Westbrook et al., 2019 0 7 0.00 [0.00; 13.10]
Sheikh Ali et al., 2008 0 9 0.00 [0.00; 10.30]
Carvalho et al., 2013 0 12 0.00 [0.00; 7.79]
Fury et al., 2013 0 14 0.00 [0.00; 6.70]
Stransky et al., 2011 0 15 0.00 [0.00; 6.27]
Braig et al., 2016 0 19 0.00 [0.00; 4.97]
Friedland et al., 2011 0 20 0.00 [0.00; 4.73]
Agarwal et al., 2011 0 21 0.00 [0.00; 4.50]
Prigge et al., 2014 0 21 0.00 [0.00; 4.50]
Dubot et al., 2018 2 22 9.09 [0.94; 24.28]
van Damme et al., 2010 1 22 4.55 [0.00; 16.91]
Morris et al., 2017 0 23 0.00 [0.00; 4.12]
Reder et al., 2019 1 24 4.17 [0.00; 15.57]
Suda et al., 2012 1 25 4.00 [0.00; 14.98]
Samstein et al., 2019 1 33 3.03 [0.00; 11.47]
Weber et al., 2003 1 33 3.03 [0.00; 11.47]
Lechner et al., 2013 2 34 5.88 [0.59; 16.11]
Ginkel et al., 2016 1 37 2.70 [0.00; 10.27]
Haft et al., 2019 0 46 0.00 [0.00; 2.07]
Zehir et al., 2018 0 52 0.00 [0.00; 1.84]
Reder et al., 2021 8 56 14.29 [6.44; 24.56]
Mirghani et al., 2018 2 62 3.23 [0.31; 9.02]
Fuji et al., 2013 0 71 0.00 [0.00; 1.35]
Wu et al., 2016 8 76 10.53 [4.68; 18.36]
Pérez Sayáns et al 2019 0 82 0.00 [0.00; 1.17]
Chau et al., 2016 0 97 0.00 [0.00; 0.99]
Bissada et al., 2013 4 123 3.25 [0.86; 7.10]
AACR GENIE V9.1 4 306 1.31 [0.34; 2.88]
Bersani et al., 2017 9 325 2.77 [1.27; 4.83]
Fixed effect model 1695 1.68 [1.12; 2.34]
Random effects model 1.49 [0.60; 2.77]
Heterogeneity: $I^2 = 62\%$, $\tau^2 = 0.0081$, $\chi^2_{31} = 80.95$ ($p < 0.01$)
Anatomic Site: Sinonasal Cavity
Fury et al., 2013 0 1 0.00 [0.00; 68.97]
Robinson et al., 2017 0 1 0.00 [0.00; 68.97]
Tetsu et al., 2010 0 1 0.00 [0.00; 68.97]
Braig et al., 2016 0 2 0.00 [0.00; 40.81]
Stransky et al., 2011 0 2 0.00 [0.00; 40.81]
Morfouace et al., 2020 0 3 0.00 [0.00; 28.74]
Chau et al., 2016 0 5 0.00 [0.00; 18.01]
Samstein et al., 2019 0 6 0.00 [0.00; 15.17]
Priestley et al., 2019 0 10 0.00 [0.00; 9.30]
Suda et al., 2012 0 11 0.00 [0.00; 8.48]
Zehir et al., 2018 0 12 0.00 [0.00; 7.79]
Morris et al., 2017 1 15 6.67 [0.01; 24.19]
Frattini et al., 2006 9 18 50.00 [27.71; 72.29]
Franchi et al., 2014 1 27 3.70 [0.00; 13.91]
Szablewski et al., 2013 12 28 42.86 [25.43; 61.26]
Dogan et al., 2017 3 30 10.00 [2.03; 23.04]
Projetti et al., 2013 2 34 5.88 [0.59; 16.11]
Lopez et al., 2012 and Garcia-Inclan et al., 2012 7 58 12.07 [5.03; 21.61]
AACR GENIE V9.1 0 80 0.00 [0.00; 1.20]
Wang et al., 2019 30 80 37.50 [27.27; 48.32]
Bornholdt et al., 2008 8 174 4.60 [2.00; 8.20]
Fixed effect model 598 8.06 [6.02; 10.38]
Random effects model 5.67 [1.33; 12.74]
Heterogeneity: $I^2 = 85\%$, $\tau^2 = 0.0549$, $\chi^2_{20} = 132.66$ ($p < 0.01$)
Anatomic Site: Salivary Gland
Tetsu et al., 2010 0 3 0.00 [0.00; 28.74]
Zehir et al., 2018 0 9 0.00 [0.00; 10.30]
Drier et al., 2016 0 10 0.00 [0.00; 9.30]
Robinson et al., 2017 0 14 0.00 [0.00; 6.70]
Khoo et al., 2017 4 15 26.67 [8.16; 51.03]
Ross et al., 2014 0 15 0.00 [0.00; 6.27]
Fonseca et al., 2015 3 17 17.65 [3.79; 38.68]
Fu et al., 2015 2 18 11.11 [1.18; 29.19]
Kang et al., 2017 0 18 0.00 [0.00; 5.24]
Priestley et al., 2019 0 19 0.00 [0.00; 4.97]
Shalmon et al., 2016 0 21 0.00 [0.00; 4.50]
Hallani et al., 2018 0 23 0.00 [0.00; 4.12]
Luk et al., 2016 0 23 0.00 [0.00; 4.12]
Stephens et al., 2013 0 24 0.00 [0.00; 3.95]
Yoo et al., 2000 2 24 8.33 [0.86; 22.39]
Dogan et al., 2019 0 25 0.00 [0.00; 3.79]
Rettig et al., 2016 0 25 0.00 [0.00; 3.79]
Choisea et al., 2015 0 29 0.00 [0.00; 3.28]
Dalin et al., 2016 0 31 0.00 [0.00; 3.07]
Morris et al., 2017 4 32 12.50 [3.50; 25.96]
Augello et al., 2006 2 33 6.06 [0.61; 16.58]
Dalin et al., 2017 0 40 0.00 [0.00; 2.38]
Kim et al., 2020 0 42 0.00 [0.00; 2.27]
Schneider et al., 2016 0 43 0.00 [0.00; 2.22]
Ho et al., 2013 0 59 0.00 [0.00; 1.62]
Mueller et al., 2020 3 63 4.76 [0.93; 11.34]
Dahse et al., 2009 1 65 1.54 [0.00; 5.93]
Mitani et al., 2016 0 65 0.00 [0.00; 1.47]
Saida et al., 2018 6 70 8.57 [3.20; 16.20]
Grunewald et al., 2015 0 84 0.00 [0.00; 1.14]
Morita et al., 2020 7 101 6.93 [2.83; 12.67]
Zehir et al., 2018 SG 1 105 0.95 [0.00; 3.69]
Cros et al., 2013 4 107 3.74 [0.99; 8.14]
Shimura et al., 2018 0 140 0.00 [0.00; 0.68]
Wang et al., 2016 0 149 0.00 [0.00; 0.64]
AACR GENIE V9.1 8 791 1.01 [0.43; 1.83]
Fixed effect model 2352 0.94 [0.59; 1.37]
Random effects model 0.98 [0.33; 1.96]
Heterogeneity: $I^2 = 67\%$, $\tau^2 = 0.0086$, $\chi^2_{35} = 104.75$ ($p < 0.01$)
Anatomic Site: Other
Morfouace et al., 2020 0 1 0.00 [0.00; 68.97]
Priestley et al., 2019 0 1 0.00 [0.00; 68.97]
Robinson et al., 2017 1 2 50.00 [0.85; 99.15]
Gauthaman et al., 2020 3 4 75.00 [27.96; 99.89]
Tetsu et al., 2010 0 5 0.00 [0.00; 18.01]
Batta et al., 2019 0 7 0.00 [0.00; 13.10]
Samstein et al., 2019 0 9 0.00 [0.00; 10.30]
Bissada et al., 2013 0 11 0.00 [0.00; 8.48]
Chau et al., 2016 0 14 0.00 [0.00; 6.70]
Ginkel et al., 2016 1 14 7.14 [0.01; 25.77]
Zehir et al., 2018 0 18 0.00 [0.00; 5.24]
Bersani et al., 2017 0 19 0.00 [0.00; 4.97]
AACR GENIE V9.1 0 21 0.00 [0.00; 4.50]
Bell et al., 2016 10 24 41.67 [23.12; 61.53]
Morris et al., 2017 3 36 8.33 [1.67; 19.41]
Popovic et al., 2010 0 39 0.00 [0.00; 2.44]
Sanchez-Fernandez et al., 2021 7 48 14.58 [6.14; 25.84]
Fixed effect model 273 4.11 [2.08; 6.78]
Random effects model 3.67 [0.30; 10.55]
Heterogeneity: $I^2 = 77\%$, $\tau^2 = 0.0552$, $\chi^2_{16} = 70.15$ ($p < 0.01$)
Fixed effect model 9609 1.31 [1.09; 1.54]
Random effects model 1.40 [0.92; 1.97]
Heterogeneity: $I^2 = 73\%$, $\tau^2 = 0.0150$, $\chi^2_{214} = 779.08$ ($p < 0.01$)
Residual heterogeneity: $I^2 = 70\%$, $\chi^2_{207} = 679.61$ ($p < 0.01$)
0 20 40 60 80 100
Prevalence (%)

NRAS Mutation in Different Anatomic Sites

Study	Cases	Total	Prevalence (%)	95% C.I.
**Anatomic Site: Hypopharynx**				
Fury et al., 2013	0	1	0.00	[0.00; 68.97]
Morris et al., 2017	0	2	0.00	[0.00; 40.81]
Westbrook et al., 2019	0	2	0.00	[0.00; 40.81]
Hedberg et al., 2016	0	3	0.00	[0.00; 28.74]
Zehir et al., 2018	0	3	0.00	[0.00; 28.74]
Priestley et al., 2019	0	5	0.00	[0.00; 18.01]
Chau et al., 2016	0	6	0.00	[0.00; 15.17]
Samstein et al., 2019	0	8	0.00	[0.00; 11.53]
Agarwal et al., 2011	0	9	0.00	[0.00; 10.30]
Stransky et al., 2011	0	9	0.00	[0.00; 10.30]
Braig et al., 2016	0	9	0.00	[0.00; 10.30]
Pérez Sayáns et al 2019	0	10	0.00	[0.00; 9.30]
Dubot et al., 2018	0	17	0.00	[0.00; 5.54]
AACR GENIE V9.1	0	29	0.00	[0.00; 3.28]
**Fixed effect model**		113	0.00	[0.00; 0.85]
**Random effects model**			0.00	[0.00; 0.85]
Heterogeneity: $I^2 = 0\%$, $\tau^2 = 0$, $\chi^2_{13} = 0$ ($p = 1.00$)				
**Anatomic Site: Larynx**				
Robinson et al., 2017	0	1	0.00	[0.00; 68.97]
Priestley et al., 2019	0	2	0.00	[0.00; 40.81]
Braig et al., 2016	1	4	25.00	[0.11; 72.04]
Hedberg et al., 2016	0	4	0.00	[0.00; 22.15]
Westbrook et al., 2019	0	7	0.00	[0.00; 13.10]
Morris et al., 2017	0	8	0.00	[0.00; 11.53]
Agarwal et al., 2011	0	13	0.00	[0.00; 7.21]
Gupta et al., 2007	0	14	0.00	[0.00; 6.70]
Samstein et al., 2019	0	14	0.00	[0.00; 6.70]
Stransky et al., 2011	0	15	0.00	[0.00; 6.27]
Zehir et al., 2018	0	16	0.00	[0.00; 5.88]
Smith et al., 2019	0	21	0.00	[0.00; 4.50]
Dubot et al., 2018	0	22	0.00	[0.00; 4.30]
Chau et al., 2016	0	24	0.00	[0.00; 3.95]
Kyurkchiyan et al., 2020	1	57	1.75	[0.00; 6.74]
Russo et al., 2006	0	81	0.00	[0.00; 1.18]
AACR GENIE V9.1	0	98	0.00	[0.00; 0.98]
Pérez Sayáns et al 2019	1	117	0.85	[0.00; 3.32]
**Fixed effect model**		518	0.16	[0.00; 0.68]
**Random effects model**			0.16	[0.00; 0.68]
Heterogeneity: $I^2 = 0\%$, $\tau^2 = 0$, $\chi^2_{17} = 9.18$ ($p = 0.93$)				
**Anatomic Site: Nasopharynx**				
Gupta et al., 2007	0	1	0.00	[0.00; 68.97]
Fury et al., 2013	0	1	0.00	[0.00; 68.97]
Westbrook et al., 2019	0	1	0.00	[0.00; 68.97]
Priestley et al., 2019	0	1	0.00	[0.00; 68.97]
Chau et al., 2016	0	7	0.00	[0.00; 13.10]
Morris et al., 2017	1	9	11.11	[0.02; 38.22]
Morfouace et al., 2020	0	10	0.00	[0.00; 9.30]
Samstein et al., 2019	1	14	7.14	[0.01; 25.77]
Zehir et al., 2018	1	17	5.88	[0.01; 21.54]
Lin et al., 2014	1	56	1.79	[0.00; 6.86]
Lin et al., 2014	0	66	0.00	[0.00; 1.45]
AACR GENIE V9.1	1	82	1.22	[0.00; 4.71]
Zhang et al., 2017	1	94	1.06	[0.00; 4.12]
Li et al., 2017	3	105	2.86	[0.55; 6.88]
Zhang et al., 2014	5	123	4.07	[1.31; 8.25]
**Fixed effect model**		587	1.85	[0.92; 3.10]
**Random effects model**			1.85	[0.92; 3.10]
Heterogeneity: $I^2 = 0\%$, $\tau^2 = 0$, $\chi^2_{14} = 13.06$ ($p = 0.52$)				
**Anatomic Site: Oral Cavity**				
Fury et al., 2013	1	1	100.00	[31.03; 100.00]
Hedberg et al., 2016	1	6	16.67	[0.04; 53.52]
Westbrook et al., 2019	0	6	0.00	[0.00; 15.17]
Tetsu et al., 2010	0	8	0.00	[0.00; 11.53]
Robinson et al., 2017	0	8	0.00	[0.00; 11.53]
Braig et al., 2016	1	12	8.33	[0.01; 29.64]
Priestley et al., 2019	0	12	0.00	[0.00; 7.79]
Gupta et al., 2007	0	20	0.00	[0.00; 4.73]
Kawamura et al., 2020	0	20	0.00	[0.00; 4.73]
Upadhyay et al., 2017	0	24	0.00	[0.00; 3.95]
Morris et al., 2017	1	26	3.85	[0.00; 14.43]
Jayaprakash et al., 2019	0	28	0.00	[0.00; 3.39]
Patel et al., 2021	0	30	0.00	[0.00; 3.17]
Cohen et al., 2011	0	37	0.00	[0.00; 2.57]
Batta et al., 2019	0	39	0.00	[0.00; 2.44]
Pickering et al., 2013	0	40	0.00	[0.00; 2.38]
Nakagaki et al., 2017	0	47	0.00	[0.00; 2.03]
Stransky et al., 2011	0	51	0.00	[0.00; 1.87]
Samstein et al., 2019	1	56	1.79	[0.00; 6.86]
Chang et al., 2010	0	58	0.00	[0.00; 1.65]
Zehir et al., 2018	1	59	1.69	[0.00; 6.52]
Chau et al., 2016	2	60	3.33	[0.33; 9.31]
Dubot et al., 2018	1	61	1.64	[0.00; 6.31]
Agarwal et al., 2011	0	75	0.00	[0.00; 1.28]
Chang et al., 2014	0	79	0.00	[0.00; 1.21]
Zanaruddin et al., 2013	0	107	0.00	[0.00; 0.89]
Sathyan et al., 2007	0	152	0.00	[0.00; 0.63]
Oikawa et al., 2016	2	220	0.91	[0.09; 2.59]
Pérez Sayáns et al 2019	0	303	0.00	[0.00; 0.32]
AACR GENIE V9.1	5	468	1.07	[0.34; 2.20]
**Fixed effect model**		2113	0.30	[0.11; 0.58]
**Random effects model**			0.26	[0.04; 0.68]
Heterogeneity: $I^2 = 35\%$, $\tau^2 = 0.0021$, $\chi^2_{29} = 44.95$ ($p = 0.03$)				
**Anatomic Site: Oropharynx**				
Robinson et al., 2017	0	2	0.00	[0.00; 40.81]
Priestley et al., 2019	0	3	0.00	[0.00; 28.74]
Westbrook et al., 2019	0	7	0.00	[0.00; 13.10]
Stanek et al., 2019	1	12	8.33	[0.01; 29.64]
Fury et al., 2013	0	14	0.00	[0.00; 6.70]
Stransky et al., 2011	0	15	0.00	[0.00; 6.27]
Braig et al., 2016	0	19	0.00	[0.00; 4.97]
Agarwal et al., 2011	0	21	0.00	[0.00; 4.50]
Dubot et al., 2018	0	22	0.00	[0.00; 4.30]
Morris et al., 2017	0	23	0.00	[0.00; 4.12]
Reder et al., 2019	1	24	4.17	[0.00; 15.57]
Samstein et al., 2019	1	33	3.03	[0.00; 11.47]
Lechner et al., 2013	0	34	0.00	[0.00; 2.80]
Haft et al., 2019	0	46	0.00	[0.00; 2.07]
Zehir et al., 2018	0	52	0.00	[0.00; 1.84]
Reder et al., 2021	7	56	12.50	[5.22; 22.34]
Mirghani et al., 2018	0	62	0.00	[0.00; 1.54]
Pérez Sayáns et al 2019	0	82	0.00	[0.00; 1.17]
Chau et al., 2016	0	97	0.00	[0.00; 0.99]
AACR GENIE V9.1	3	306	0.98	[0.19; 2.39]
Bersani et al., 2017	4	325	1.23	[0.32; 2.72]
**Fixed effect model**		1255	0.65	[0.28; 1.16]
**Random effects model**			0.48	[0.06; 1.31]
Heterogeneity: $I^2 = 46\%$, $\tau^2 = 0.0040$, $\chi^2_{20} = 37.1$ ($p = 0.01$)				
**Anatomic Site: Sinonasal Cavity**				
Tetsu et al., 2010	0	1	0.00	[0.00; 68.97]
Fury et al., 2013	0	1	0.00	[0.00; 68.97]
Robinson et al., 2017	0	1	0.00	[0.00; 68.97]
Stransky et al., 2011	0	2	0.00	[0.00; 40.81]
Braig et al., 2016	0	2	0.00	[0.00; 40.81]
Morfouace et al., 2020	0	3	0.00	[0.00; 28.74]
Chau et al., 2016	0	5	0.00	[0.00; 18.01]
Samstein et al., 2019	0	6	0.00	[0.00; 15.17]
Priestley et al., 2019	0	10	0.00	[0.00; 9.30]
Zehir et al., 2018	0	12	0.00	[0.00; 7.79]
Morris et al., 2017	0	15	0.00	[0.00; 6.27]
Dogan et al., 2017	0	30	0.00	[0.00; 3.17]
AACR GENIE V9.1	1	80	1.25	[0.00; 4.83]
**Fixed effect model**		168	0.28	[0.00; 1.65]
**Random effects model**			0.28	[0.00; 1.65]
Heterogeneity: $I^2 = 0\%$, $\tau^2 = 0$, $\chi^2_{12} = 2.1$ ($p = 1.00$)				
**Anatomic Site: Salivary Gland**				
Tetsu et al., 2010	1	3	33.33	[0.25; 85.58]
Zehir et al., 2018	0	9	0.00	[0.00; 10.30]
Drier et al., 2016	0	10	0.00	[0.00; 9.30]
Robinson et al., 2017	0	14	0.00	[0.00; 6.70]
Ross et al., 2014	0	15	0.00	[0.00; 6.27]
Khoo et al., 2017	4	15	26.67	[8.16; 51.03]
Fonseca et al., 2015	1	17	5.88	[0.01; 21.54]
Fu et al., 2015	0	18	0.00	[0.00; 5.24]
Kang et al., 2017	0	18	0.00	[0.00; 5.24]
Priestley et al., 2019	0	19	0.00	[0.00; 4.97]
Luk et al., 2016	1	23	4.35	[0.00; 16.21]
Hallani et al., 2018	0	23	0.00	[0.00; 4.12]
Stephens et al., 2013	0	24	0.00	[0.00; 3.95]
Rettig et al., 2016	1	25	4.00	[0.00; 14.98]
Dogan et al., 2019	0	25	0.00	[0.00; 3.79]
Choisea et al., 2015	0	29	0.00	[0.00; 3.28]
Dalin et al., 2016	0	31	0.00	[0.00; 3.07]
Morris et al., 2017	0	32	0.00	[0.00; 2.97]
Hsieh et al., 2020	8	33	24.24	[11.39; 40.07]
Dalin et al., 2017	0	40	0.00	[0.00; 2.38]
Kim et al., 2020	0	42	0.00	[0.00; 2.27]
Ho et al., 2013	1	59	1.69	[0.00; 6.52]
Mueller et al., 2020	0	63	0.00	[0.00; 1.52]
Mitani et al., 2016	0	65	0.00	[0.00; 1.47]
Saida et al., 2018	0	70	0.00	[0.00; 1.37]
Grunewald et al., 2015	2	84	2.38	[0.23; 6.70]
Morita et al., 2020	0	101	0.00	[0.00; 0.95]
Zehir et al., 2018 SG	1	105	0.95	[0.00; 3.69]
Cros et al., 2013	2	107	1.87	[0.18; 5.28]
Shimura et al., 2018	0	140	0.00	[0.00; 0.68]
Wang et al., 2016	0	149	0.00	[0.00; 0.64]
AACR GENIE V9.1	5	791	0.63	[0.20; 1.30]
**Fixed effect model**		2199	0.46	[0.22; 0.79]
**Random effects model**			0.51	[0.11; 1.22]
Heterogeneity: $I^2 = 57\%$, $\tau^2 = 0.0056$, $\chi^2_{31} = 72.87$ ($p < 0.01$)				
**Anatomic Site: Other**				
Morfouace et al., 2020	0	1	0.00	[0.00; 68.97]
Priestley et al., 2019	0	1	0.00	[0.00; 68.97]
Robinson et al., 2017	0	2	0.00	[0.00; 40.81]
Tetsu et al., 2010	0	5	0.00	[0.00; 18.01]
Batta et al., 2019	0	7	0.00	[0.00; 13.10]
Samstein et al., 2019	0	9	0.00	[0.00; 10.30]
Chau et al., 2016	0	14	0.00	[0.00; 6.70]
Zehir et al., 2018	1	18	5.56	[0.00; 20.42]
Bersani et al., 2017	0	19	0.00	[0.00; 4.97]
AACR GENIE V9.1	0	21	0.00	[0.00; 4.50]
Bell et al., 2016	2	24	8.33	[0.86; 22.39]
Morris et al., 2017	0	36	0.00	[0.00; 2.64]
Popovic et al., 2010	2	39	5.13	[0.51; 14.13]
Sanchez-Fernandez et al., 2021	2	48	4.17	[0.41; 11.57]
**Fixed effect model**		244	1.51	[0.37; 3.42]
**Random effects model**			1.46	[0.31; 3.45]
Heterogeneity: $I^2 = 5\%$, $\tau^2 = 0.0008$, $\chi^2_{13} = 13.72$ ($p = 0.39$)				
**Fixed effect model**		**7197**	**0.49**	**[0.35; 0.67]**
**Random effects model**			**0.46**	**[0.27; 0.70]**
Heterogeneity: $I^2 = 27\%$, $\tau^2 = 0.0020$, $\chi^2_{156} = 212.91$ ($p < 0.01$)				
Residual heterogeneity: $I^2 = 23\%$, $\chi^2_{149} = 192.97$ ($p < 0.01$)				

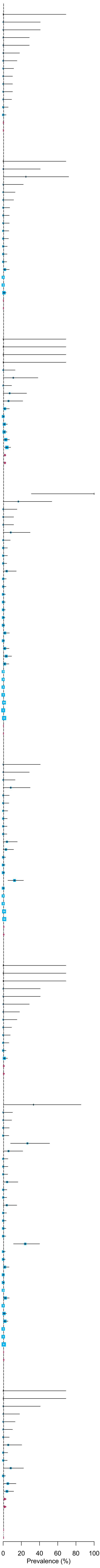

Figure S4: Association between RAS Mutations and Disease Stage/Grade

An odds ratio analysis of the association between tumor grade and KRAS or NRAS mutations; no statistically significant correlations were found.

KRAS

Study	High Grade Events	High Grade Total	Low Grade Events	Low Grade Total	OR	95%-CI	Weight (fixed)	Weight (random)
Lin et al., 2014	1	1	0	1	9.00	[0.10; 831.78]	1.4%	3.0%
Hedberg et al., 2016	1	1	0	1	9.00	[0.10; 831.78]	1.4%	3.0%
Li et al., 2017	0	1	1	1	0.11	[0.00; 10.27]	1.4%	3.0%
Reder et al., 2019	0	1	1	1	0.11	[0.00; 10.27]	1.4%	3.0%
Pérez Sayáns et al 2019	1	1	0	1	9.00	[0.10; 831.78]	1.4%	3.0%
Oikawa et al.,2016	1	1	0	1	9.00	[0.10; 831.78]	1.4%	3.0%
Bruckman et al., 2010	1	1	0	1	9.00	[0.10; 831.78]	1.4%	3.0%
Fury et al., 2013	1	1	0	1	9.00	[0.10; 831.78]	1.4%	3.0%
Franchi et al., 2014	0	1	1	1	0.11	[0.00; 10.27]	1.4%	3.0%
Akagi et al., 2019	0	1	1	1	0.11	[0.00; 10.27]	1.4%	3.0%
Morris et al., 2017	2	2	0	2	25.00	[0.34; 1831.59]	1.5%	3.1%
Dubot et al., 2018	1	2	1	2	1.00	[0.02; 50.40]	1.8%	3.4%
Fu et al., 2015	2	2	0	2	25.00	[0.34; 1831.59]	1.5%	3.1%
Dogan et al., 2017	2	3	1	3	4.00	[0.13; 119.23]	2.4%	3.9%
Suda et al., 2012	3	3	0	3	49.00	[0.74; 3236.99]	1.6%	3.2%
Das et al., 2000	4	4	0	4	81.00	[1.30; 5046.33]	1.6%	3.3%
Khoo et al., 2017	2	4	2	4	1.00	[0.06; 15.99]	3.6%	4.5%
Weber et al., 2003	2	4	2	4	1.00	[0.06; 15.99]	3.6%	4.5%
Saida et al., 2018	1	6	5	6	0.04	[0.00; 0.83]	3.0%	4.2%
ORCA ICGC	6	6	0	6	169.00	[2.89; 9875.38]	1.7%	3.3%
Morita et al., 2020	4	7	3	7	1.78	[0.21; 14.77]	6.3%	5.2%
Sanchez-Fernandez et al., 2021	1	7	6	7	0.03	[0.00; 0.55]	3.1%	4.3%
Lopez et al., 2012 and Garcia-Inclan et al., 2012	0	7	7	7	0.00	[0.00; 0.25]	1.7%	3.3%
Bissada et al., 2013	7	7	0	7	225.00	[3.93; 12894.87]	1.7%	3.3%
Wu et al., 2016	15	18	3	18	25.00	[4.33; 144.30]	9.1%	5.6%
Wang et al., 2019	11	27	16	27	0.47	[0.16; 1.40]	23.8%	6.2%
Gauthaman et al., 2020	28	34	6	34	21.78	[6.26; 75.78]	18.0%	6.0%
**Fixed effect model**		**153**		**153**	**2.48**	**[1.46; 4.22]**	**100.0%**	**--**
**Random effects model**					**2.41**	**[0.85; 6.86]**	**--**	**100.0%**

Heterogeneity: $I^2 = 67\%$, $\tau^2 = 4.3095$, $\chi^2_{26} = 78.52$ ($p < 0.01$)

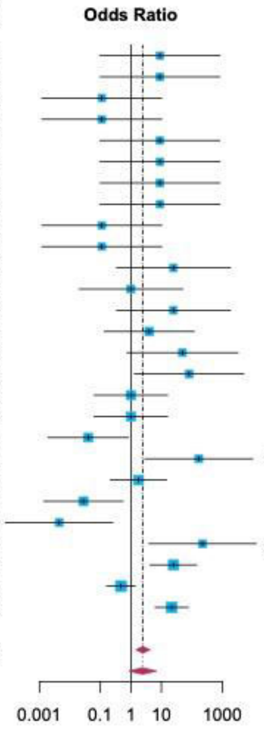

NRAS

Study	High Grade Events	High Grade Total	Low Grade Events	Low Grade Total	OR	95%-CI	Weight (fixed)	Weight (random)
Lin et al., 2014	1	1	0	1	9.00	[0.10; 831.78]	1.3%	6.5%
Luk et al., 2016	0	1	1	1	0.11	[0.00; 10.27]	11.7%	6.5%
Hedberg et al., 2016	1	1	0	1	9.00	[0.10; 831.78]	1.3%	6.5%
Reder et al., 2019	0	1	1	1	0.11	[0.00; 10.27]	11.7%	6.5%
Sanchez-Fernandez et al., 2021	1	1	0	1	9.00	[0.10; 831.78]	1.3%	6.5%
Dubot et al., 2018	1	1	0	1	9.00	[0.10; 831.78]	1.3%	6.5%
Pickering et al., 2014	0	1	1	1	0.11	[0.00; 10.27]	11.7%	6.5%
Morris et al., 2017	2	2	0	2	25.00	[0.34; 1831.59]	0.9%	7.0%
Li et al., 2017	2	2	0	2	25.00	[0.34; 1831.59]	0.9%	7.0%
ORCA ICGC	2	2	0	2	25.00	[0.34; 1831.59]	0.9%	7.0%
Oikawa et al.,2016	2	2	0	2	25.00	[0.34; 1831.59]	0.9%	7.0%
Khoo et al., 2017	1	4	3	4	0.11	[0.00; 2.73]	23.5%	10.4%
Hsieh et al., 2020	3	8	5	8	0.36	[0.05; 2.73]	32.6%	16.3%
**Fixed effect model**		**27**		**27**	**1.52**	**[0.68; 3.41]**	**100.0%**	**--**
**Random effects model**					**1.91**	**[0.51; 7.19]**	**--**	**100.0%**

Heterogeneity: $I^2 = 31\%$, $\tau^2 = 1.7314$, $\chi^2_{12} = 17.27$ ($p = 0.14$)

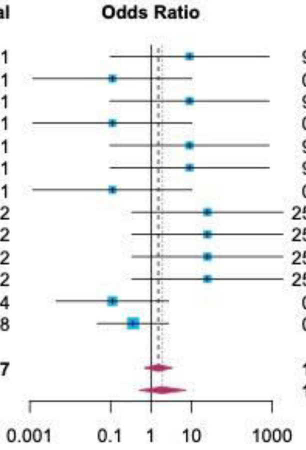

Figure S5: Association between RAS Mutations and HPV status

An odds ratio analysis of the association between human papillomavirus infection status and HRAS or NRAS mutations; no statistically significant correlations were found.

HRAS

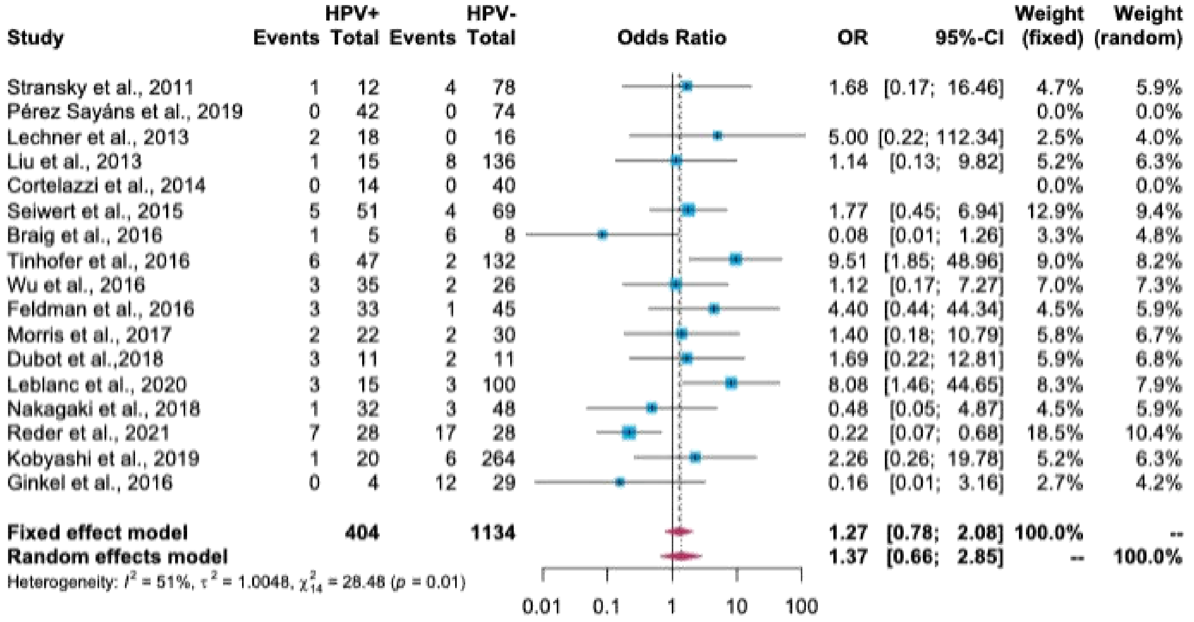

NRAS

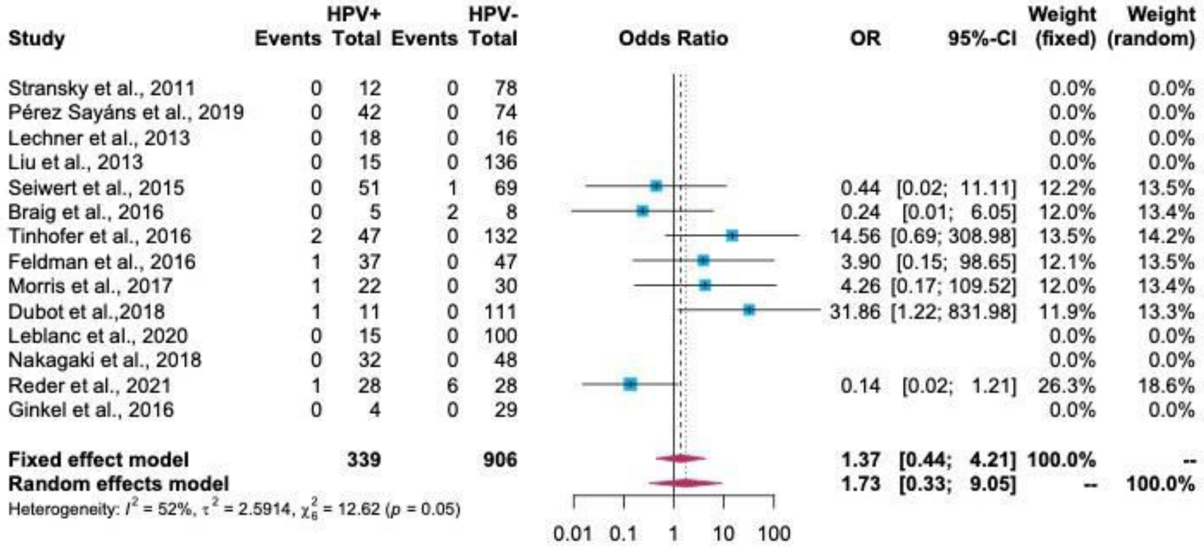

Supplement: Supplementary file 2 [file DataSheet_2.pdf]
